# 3α,5α-Cyclocholestan-6β-yl ethers as donors of the cholesterol moiety for the electrochemical synthesis of cholesterol glycoconjugates

**DOI:** 10.3762/bjoc.11.16

**Published:** 2015-01-26

**Authors:** Aneta M Tomkiel, Adam Biedrzycki, Jolanta Płoszyńska, Dorota Naróg, Andrzej Sobkowiak, Jacek W Morzycki

**Affiliations:** 1 Institute of Chemistry, University of Białystok, ul. Ciołkowskiego 1K, 15-245 Białystok, Poland; 2 Faculty of Chemistry, Rzeszów University of Technology, P.O. Box 85, 35-959 Rzeszów, Poland

**Keywords:** cholesterol, electrochemical oxidation, glycosylation, i-cholesteryl ethers, i-steroids

## Abstract

3α,5α-Cyclocholestan-6β-yl alkyl and aryl ethers were proved to be efficient cholesteryl donors in the electrochemical synthesis of glycoconjugates. 3α,5α-Cyclocholestan-6β-ol (i-cholesterol) and its *tert*-butyldimethylsilyl ether can also be used for this purpose. The i-cholesterol derivatives show similar reactivities to those of previously studied 3α,5α-cyclocholestan-6β-thioethers.

## Introduction

**Scheme 1:**
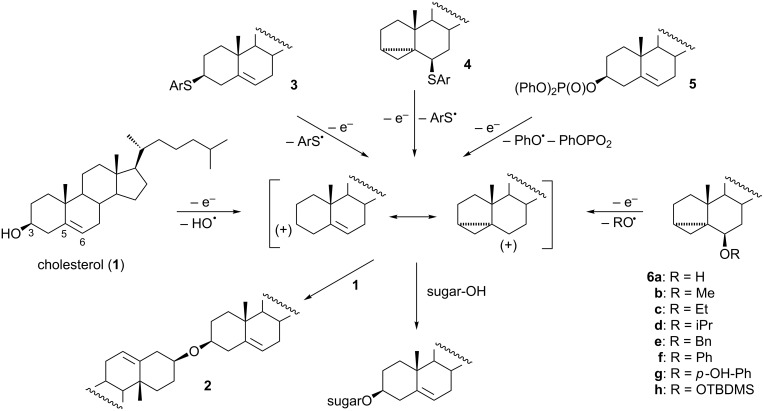
Synthesis of glycoconjugates from different cholesteryl donors.

We have recently elaborated an electrochemical method for the preparation of glycosides and glycoconjugates from 3β-hydroxy-Δ^5^-steroids (sterols). The method consists of electrooxidation of a proper cholesterol derivative in the presence of an unactivated sugar with a free hydroxy group at the anomeric position (formation of glycosides) or at any other position (preferentially a primary position) that leads to sterol glycoconjugates. Initially, the reaction of cholesterol (**1**) with various sugars was studied. During anodic oxidation of cholesterol in dichloromethane (the choice of solvent is crucial as the reaction course may be different in other solvents) [1], splitting of the carbon–oxygen bond in an intermediate radical cation occurs, thus leading to a mesomerically stabilized homoallylic carbocation and a hydroxyl radical (Scheme 1) [2]. However, the glycosylation reaction was not very efficient due to competition between the sugar alcohol and cholesterol for the carbocation [3]. If cholesterol wins the competition, the dimer **2** (dicholesteryl ether) is formed. A large excess of sugar was used to avoid this undesired side reaction. In our further study, instead of cholesterol, cholest-5-en-3β-yl and 3α,5α-cyclocholestan-6β-yl thioethers (**3** and **4**) were applied as cholesteryl donors [4]. Of the two, the latter appeared to be much more efficient in cholesteryl transfer to the sugar moiety. The problem, however, with 6β-steroidal thioethers is that they are not easily accessible.

Therefore, we searched for the best leaving group at C-3. A series of cholesterol derivatives was tested and, finally, cholesteryl diphenylphosphate (**5**) was selected as the best cholesteryl donor [5]. Apart from these cholesterol derivatives, also simple alkyl or aryl ethers (**6b**–**g**) of 3α,5α-cyclocholestan-6β-ol (**6a**) (i-cholesterol) are readily available by solvolysis of cholesteryl *p*-tosylate in the corresponding alcohol under buffered conditions, while *tert*-butyldimethylsilyl ether **6h** can be prepared by silylation of i-cholesterol **6a** with TBDMSCl. Now we report the results of our study on the application of these ethers as cholesteryl donors in electrochemical glycosylation reactions.

## Results and Discussion

The cyclosteroid (i-steroid) rearrangement is a well-known steroid reaction [6]. The solvolysis of cholesteryl *p*-tosylate proceeds via the S_N_1 mechanism with the formation of a mesomerically stabilized carbocation. The addition of a nucleophile (alcohol, water, etc.) may occur either to C-3 or C-6, depending on the reaction conditions. In both cases the nucleophile is attached only from the upper side (β) of cholesterol due to stereo-electronic reasons. Under buffered conditions the addition is irreversible and leads to 3α,5α-cyclocholestan-6β-substituted products in excess. The addition to C-6 is faster (the kinetic product is formed) since the mesomeric carbocation is more positively charged in this position than in C-3. However, under acidic conditions the reaction becomes reversible and the 3β-substituted product, which is more stable (the thermodynamic product), is exclusively formed. The i-steroid ethers are frequently prepared for simultaneous protection of both 3β-ol and Δ^5^ groups in sterols. The cycloreversion that occurs under acidic conditions allows to recover sterol functions.

We thought that such highly energetic i-steroid ethers would easily generate the mesomeric carbocation during electrochemical oxidation by cleavage of the carbon–oxygen bond in an intermediate radical-cation. For this reason, i-cholesteryl ethers seemed to be suitable donors of the cholesterol moiety for the electrochemical synthesis of cholesterol glycoconjugates.

A series of i-cholesterol derivatives **6b–h** was prepared including alkyl, aryl, and silyl ethers. Most of the compounds were prepared by solvolysis of cholesteryl *p*-tosylate in an appropriate alcohol (neat or mixed with dioxane) in the presence of potassium acetate as a buffer. TBDMS ether **6h** was obtained by silylation of i-cholesterol **6a** with TBDMSCl in DMF in the presence of imidazole and DMAP.

**Figure 1:**
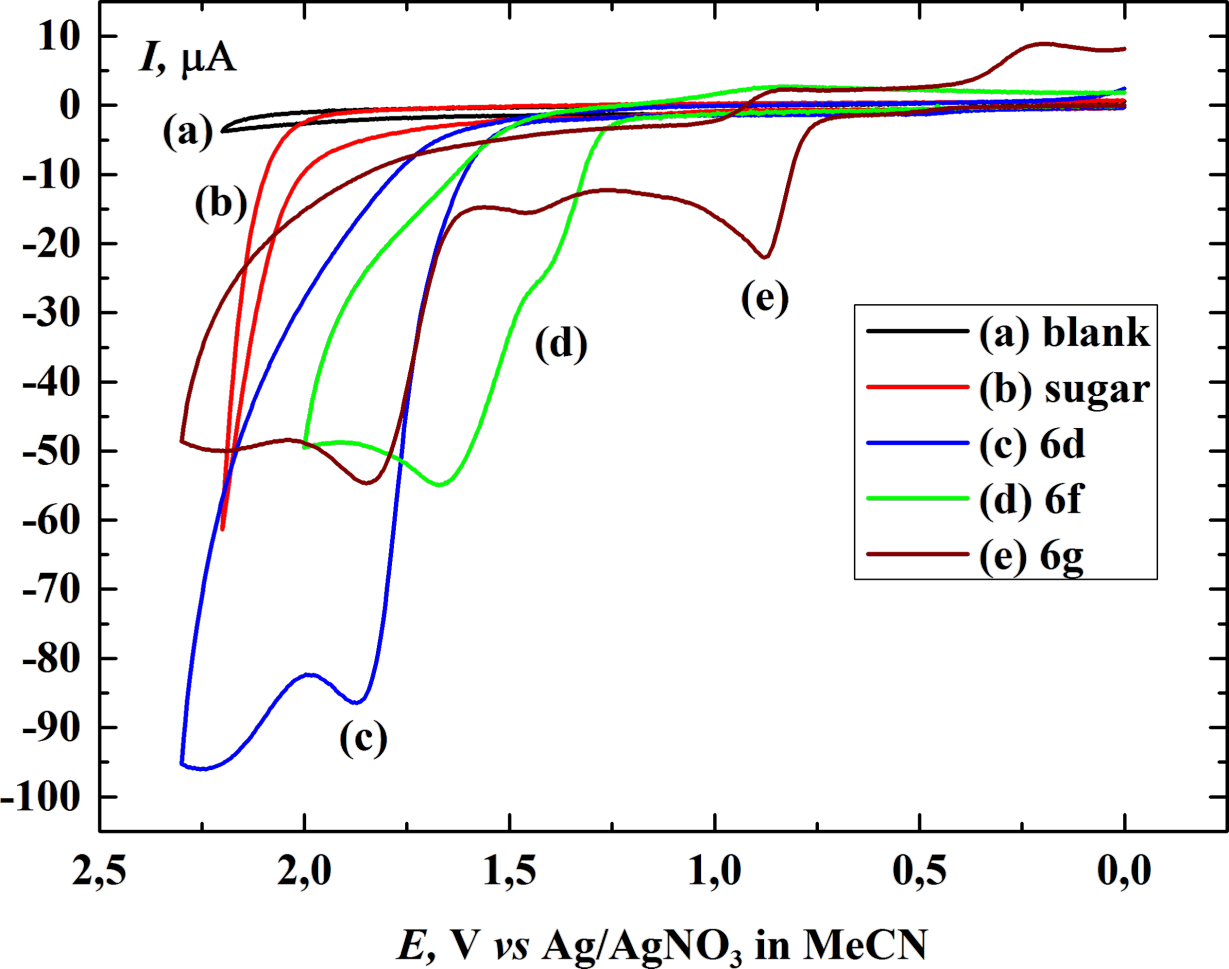
Cyclic voltammograms registered in 0.2 M tetrabutylammonium tetrafluoroborate (TBABF_4_) in dichloromethane on a platinum electrode (area, 0.008 cm^2^) of (a) the supporting electrolyte (black), (b) 1,2:3,4-di-*O*-isopropylidene-α-D-galactopyranose (**7**) (red), (c) 6β-isopropyloxy-3α,5α-cyclocholestane (**6d**), (blue) (d) 6β-phenyloxy-3α,5α-cyclocholestane (**6f**) (green) and (e) 6β-(4-hydroxyphenyloxy)-3α,5α-cyclocholestane (**6g**) (brown). Concentrations of all compounds are equal to 5 mM, scan rate 1 V s^−1^. Potentials were measured vs Ag/0.1 M AgNO_3_ in acetonitrile at room temperature.

All of these compounds proved to be electrochemically active. Cyclic voltammograms measured in dichloromethane on a platinum electrode indicated that the main oxidation peak for the substances occurred within 1.8–2.0 V (vs Ag/AgNO_3_ in MeCN). The shapes of the voltammograms are similar (except **6f** and **6g**) to the one for 6β-isopropyloxy-3α,5α-cyclocholestane (**6d**), which is presented in Figure 1 (curve c, blue). The cyclic voltammogram for 6β-phenyloxy-3α,5α-cyclocholestane (**6f**) shows an additional anodic peak at 1.45 V (curve d, green) and the cyclic voltammogram for 6β-(4-hydroxyphenyloxy)-3α,5α-cyclocholestane (**6g**) shows two additional anodic peaks at 0.88 V and 1.45 V (curve e, brown). The existence of these peaks is probably connected with electrooxidation of the substituents. It is worth to notice that in the case of **6f** and **6g** the oxidation currents are approximately equal to the half of oxidation current of **6d** and also other investigated compounds. This is probably caused by the electrode blocking due to an adsorption of the oxidation products. The fact that the electrochemical oxidation of **6f** and **6g** starts at more negative potentials in comparison to the other investigated compounds supports this explanation. Moreover, during subsequent voltammetric cycles of **6d** the oxidation current is decreased to almost the half of the current registered in the first scan. In addition the second peak for **6d** exhibits the most positive value among ethers investigated. This observation is difficult to explain without further investigations. Figure 1 also presents the voltammogram of a model sugar alcohol – 1,2:3,4-di-*O*-isopropylidene-α-D-galactopyranose (**7**) (curve b, red line), which indicates that the substrate is electrochemically inactive within the applied potential region. The readily available α-D-galactopyranose derivative **7** was chosen as a model sugar since the anomeric and secondary hydroxy groups in this compound are protected as diacetonide and the remaining primary hydroxy group is highly reactive.

**Scheme 2:**
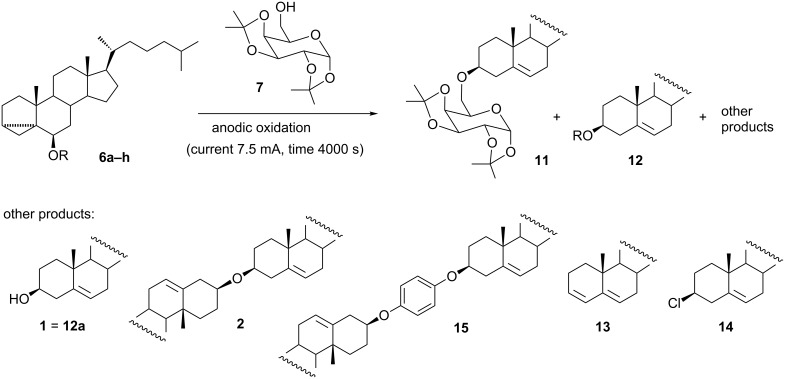
Electrochemical reaction of 3α,5α-cyclocholestan-6β-yl ethers **6a–h** with 1,2:3,4-di-*O*-isopropylidene-D-galactopyranose (**7**).

The results of preparative electrolysis of compounds **6a**–**h** in the presence of 1,2:3,4-di-*O*-isopropylidene-α-D-galactopyranose (**7**) are shown in Scheme 2. In all of the experiments the ratio of sugar:cholesteryl donor was close to 1:1. The electrochemical reaction conditions were the same as those applied in our previous paper for the preparation of cholesterol glycoconjugates by using cholesteryl donors with an activating group at C-3 (such as diphenylphosphate **5**) [5].

The glycosylation reactions leading to **11** were accompanied by isomerization of substrates to analogous cholesterol 3β-derivatives **12** (Table 1). Compounds **12** proved much less electrochemically active or not active at all.

**Table 1:** Electrochemical oxidation^a^ of 3α,5α-cyclocholestan-6β-yl ethers **6a–h** in the presence of 1,2:3,4-di-*O*-isopropylidene-α-D-galactopyranose (**7**).

Substrate **6**	3β-*O*-(1’,2’:3’,4’-di-*O*-isopropylidene-D-galactopyranos-6’-yl)-cholest-5-ene (**11**) (yield)	Isomerization product **12** (yield)	Other steroidal product (yield)

**6a**	47%	8%^b^	**2** (17), **14** (3%), **13** (2%)
**6b**	40%	24%	**13** (2%), **1** (1%)
**6c**	51%	45%	**13** (2%), **14** (1%)
**6d**	41%	34%	**1** (2%), **2** (2%)
**6e**	50%	40%	**1** (trace)
**6f**	58%	22%	**1** (4%), **13** (1%), **14** (1%)
**6g**	9%	12%	**15** (18%)
**6h**	52%	1%	**13** (9%), **1** (6%), **2** (5%)

^a^Electrochemical reaction conditions given in the Experimental section. ^b^Compound **12a** = **1** (cholesterol).

The electrochemical reaction of i-cholesterol (**6a**) with 1,2:3,4-di-*O*-isopropylidene-α-D-galactopyranose (**7**) afforded product **11** in 47% yield in addition to the product of isomerization (cholesterol (**1**), 8%) and dicholesteryl ether (**2**) (17%). Minor amounts of diene **13** and cholesteryl chloride (**14**) were also formed.

In the reactions of i-cholesterol alkyl ethers (methyl **6b**, ethyl, **6c**, isopropyl **6d**, and benzyl **6e**), cholesterol glycoconjugate **11** was also formed in 40%, 51%, 41%, and 50% yield, respectively. Glycoconjugate **11** was accompanied by substantial amounts of isomerization products **12** (24–45%) and tiny amounts of other products (cholesterol (**1**), dicholesteryl ether (**2**), diene **13**, and cholesteryl chloride **14**).

The best yield (58%) of cholesterol glycoconjugate **11** was achieved with 3α,5α-cyclocholestan-6β-yl phenyl ether (**6f**). The reaction was relatively clean; the isomerization product, i.e., cholesteryl phenyl ether (**12f**), was obtained in 22% yield. It should be emphasized that the yield of **11** was even better than those obtained in analogous reactions of 3α,5α-cyclocholestan-6β-yl phenyl thioether (40%) and 3α,5α-cyclocholestan-6β-yl *p*-tolyl thioether (52%) which we had previously studied [4]. Now it seems to be clear that high efficiency in the generation of the mesomeric carbocation may be attributed to the 3α,5α-cyclocholestan-6β-yl moiety rather than to the presence of an arylthiol group at C-3 or C-6. This conclusion is supported by the fact that cholesteryl phenyl thioether proved to be a rather poor cholesteryl donor for the electrochemical reaction (12% yield).

In contrast to the above, the reaction of 4-hydroxyphenyl i-cholesteryl ether **6g** was messy and afforded only 9% of the desired glycoconjugate **11**. The isomerization product, i.e., 4-hydroxyphenyl cholesteryl ether **12g**, was formed in 12% yield, while the major reaction product (18%) was 1,4-phenylene dicholesteryl diether (**15**).

The difference in reactivity between **6f** and **6g** is rather surprising. Both compounds are oxidized at relatively low potentials, which suggests that the mesomeric carbocation is formed more easily than in the case of the other investigated compounds. Indeed, the best yield of glycosylation obtained for 3α,5α-cyclocholestan-6β-yl phenyl ether (**6f**) as a substrate supports the hypothesis. On the other hand, the reason for the low yield observed for 4-hydroxyphenyl i-cholesteryl ether (**6g**) can be attributed to the electrochemical oxidation of the phenol type substituent, which is responsible for an additional peak at low potentials. The process is usually irreversible, resulting from the fast deprotonation of the primarily generated radical cation [7]. The phenoxy radical formed can be further oxidized to the phenoxenium ion, which reacts with nucleophiles. However, since the phenolic OH group is not sterically shielded here, competitive oligo- and polymerization reactions may also occur. Therefore, despite the low oxidation potential of **6g**, the side reactions account for a low yield of the desired product.

Relatively satisfactory results were obtained with i-cholesterol TBDMS ether **6h**. The glycoconjugate **11** was obtained in 52% yield with only a trace amount of the isomerization product **12h**. The major byproduct in this case was diene **13** (9%).

**Scheme 3:**
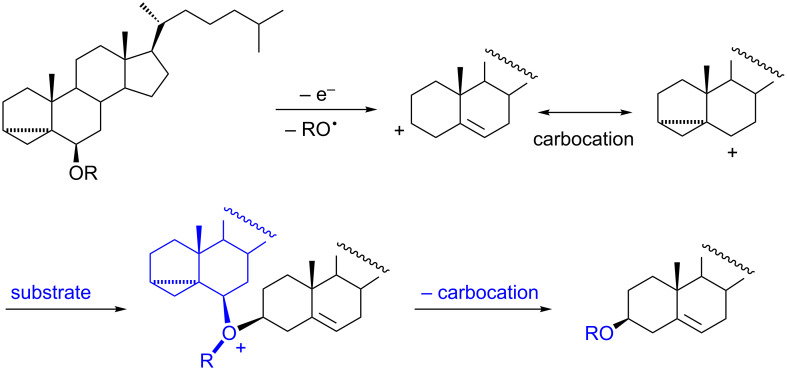
Plausible mechanism of isomerization.

The occurrence of the isomerization process during electrochemical oxidation of i-cholesterol ethers is rather surprising. We proved that without electrochemical activation the glycosylation or isomerization processes do not take place under the reaction conditions. The likely mechanism of isomerization is shown in Scheme 3. The proposed formation of disteroidal oxonium ions accounts for an alkyl (aryl) group transfer from C-6 to C-3. Interestingly, the isomerization itself is not an electrochemical reaction. Electrooxidation is needed only to initiate the whole process, i.e., to generate the homoallylic carbocation; then the chain process occurs. The blank experiments (electrochemical reactions without the presence of sugar) were carried out to establish the approximate isomerization rates. The concentration of 3α,5α-cyclocholestan-6β-yl alkyl ether (methyl or ethyl) in the reaction mixture has fallen by half in less than 15 minutes, i.e., after that time the 3- and 6-substituted ether concentrations were approximately equal.

Even in the case of i-cholesteryl phenyl ether (**6f**), fast isomerization to **12f** was observed. It is worth noting that no isomerization occurred for i-cholesterol TBDMS ether (**6h**), probably due to steric reasons.

A limitation of the glycosylation process are the consecutive reactions of glycoconjugate **11** at a growing voltage and its limited stability under electrolysis conditions. The major decomposition product is 3β-O-(3’,4’-*O*-isopropylidene-α-D-galactopyranos-6’-yl)-cholest-5-ene, i.e., compound **11** loses one of the *O*-isopropylidene groups upon prolonged electrolysis. Although the electrochemical overoxidation of glycoconjugate **11** seems to be a minor problem at the initial steps due to a relatively large difference in the oxidation potentials and concentrations between substrates (sterol donors, sugar alcohol) and the resulting glycoconjugate, the possibility that side processes will occur increases as the anode potential is more positive and the glycoconjugate concentration grows.

A series of blank experiments proved that the electrochemical activation of i-steroidal ethers **6a**–**h** is necessary for their reactions with sugar **7**. No coupling occurred when chemical promoters were attempted.

## Conclusion

3α,5α-Cyclocholestan-6β-yl ethers are excellent cholesteryl donors for the electrochemical synthesis of cholesterol glycoconjugates. All of the tested compounds proved efficient in this respect, although the best yields were obtained with ethyl, benzyl, phenyl, and TBDMS ether. The side reaction is isomerization to the much less reactive cholesteryl ethers. This undesired reaction may be partially suppressed by using 3α,5α-cyclocholestan-6β-yl phenyl ether or completely stopped by employing TBDMS ether.

## Experimental

Cyclic voltammograms were recorded with iR compensation at 25 °C using a three-electrode potentiostat (Princeton Applied Research, model Parstat 2273). The experiments were conducted in a 3 mL electrochemical cell with an argon-purge system. The working electrode was a Bioanalytical Systems platinum inlay (1 mm in diameter), the auxiliary electrode was a platinum mesh (contained in a glass tube with a medium porosity glass frit), and the reference electrode was Ag/0.1 M AgNO_3_ in acetonitrile. The potential of the electrode vs the ferrocene/ferricinium ion reference couple is equal to −37 mV [8]. The reference electrode was contained in a Pyrex tube with a cracked softglass tip which was placed inside a Luggin capillary. Before each experiment, the working electrode was polished using Buehler Micropolish Alumina Gamma 3B and a Buehler Microcloth polishing cloth, rinsed with dichloromethane and dried. In all of the measurements, 0.2 M solution of tetrabutylammonium tetrafluoroborate (TBABF_4_) from Aldrich in dichloromethane was used as a supporting electrolyte.

The preparative electrolyses were performed with a potentiostat/galvanostat (Princeton Applied Research, model Parstat 2273) under galvanostatic conditions using a current that was equal in a typical experiment to 7.5 mA and a reaction time of 4000 s. The current applied was the maximum current available for the electrolysis set-up being used (power supply and ohmic resistance). During the electrolysis the potential of the anode was monitored and the process was stopped when the potential reached the value of +2.3 V vs Ag/0.1 M AgNO_3_ to avoid the occurrence of undesired oxidation processes. The reactions were also monitored by TLC and stopped when no further increase in the concentration of the glycosylation products was observed. A divided H-cell was used in which the cathodic and anodic compartments (3.5 mL of electrolyte each) were separated by a glass frit. In all measurements, 0.1 M solution of tetrabutylammonium tetrafluoroborate (TBABF_4_) from Aldrich in dichloromethane was used as a supporting electrolyte. The steroid (0.30 mmol) and sugar (0.36 mmol) substrates were introduced into the anodic compartment together with 0.3 g of 3 Å molecular sieves added to eliminate traces of water, whereas anionite (1.5–2 g, Dowex 2 × 8, 200–400 mesh, perchlorate form) was placed in the cathodic compartment to eliminate chloride ions that are formed by the reduction of dichloromethane. The solutions in both compartments were stirred during electrolysis and, additionally, a continuous flow of argon was applied in the anodic compartment. A platinum mesh was used as a cathode and a platinum plate (2 × 1.5 cm) was used as an anode. All measurements were performed at 25 °C.

The sugar (1,2:3,4-di-*O*-isopropylidene-α-D-galactopyranose; **7**) [9] and steroidal substrates, 3α,5α-cyclocholestan-6β-ol (i-cholesterol; **6a**) [10], 6β-methoxy-3α,5α-cyclocholestane (**6b**) [11], and 6β-ethoxy-3α,5α-cyclocholestane (**6c**) [12], were prepared according to known procedures.

Melting points were determined on a Toledo Mettler-MP70 apparatus. ^1^H and ^13^C NMR (400 and 100 MHz, respectively) spectra were recorded on a Bruker Avance II spectrometer in CDCl_3_ solutions with TMS as the internal standard (only selected signals in the ^1^H NMR spectra are reported in Supporting Information File 1; sugar protons are marked with the ‘prime’ index). Infrared spectra were recorded on a Nicolet series II Magna-IR 550 FTIR spectrometer in chloroform solutions. Mass spectra were recorded at 70 eV with a time-of-flight (TOF) AMD-604 spectrometer with electrospray ionization (ESI) or AutoSpec Premier (Waters) (EI).

Merck Silica Gel 60, F 256 TLC aluminum sheets were applied for thin-layer chromatographic analysis. For a visualization of the products, a 5% solution of phosphomolybdic acid in ethanol was used. The reaction products were separated by column chromatography performed on a 70–230 mesh silica gel (J. T. Baker).

**Typical electrochemical experiment. Anodic oxidation of 6β-phenyloxy-3α,5α-cyclocholestane (6f) in the presence of 1,2:3,4-di-*O*-isopropylidene-D-galactopyranose (7):** 6β-Phenyloxy-3α,5α-cyclocholestane (138 mg, 0.30 mmol) and 1,2:3,4-di-*O*-isopropylidene-D-galactopyranose (94 mg, 0.36 mmol) were dissolved in a 0.1 M solution of tetrabutylammonium tetrafluoroborate in dichloromethane (3.5 mL) and introduced into the anodic compartment together with 0.5 g 3 Å molecular sieves to eliminate traces of water. The same supporting electrolyte was placed in the cathodic compartment with an anionite (2 g, Dowex 2 × 8, 200–400 mesh, perchlorate form) added. Preparative electrolysis was carried out in a divided H-cell in which the cathodic and anodic compartments (3.5 mL of electrolytes each) were separated by a glass frit under galvanostatic conditions. A direct current 7.5 mA was run for 4000 s. A platinum mesh was used as a cathode and a platinum plate (2 × 1.5 cm) was used as an anode. Ag/0.1 M AgNO_3_ in an acetonitrile electrode was used as a reference. When the electrolysis was completed, the solvent was removed from the reaction mixture and the products were separated by silica gel column chromatography. The hexane elution afforded diene **13** (1 mg, 1%) and cholesteryl chloride **14** (1 mg, 1%). With the hexane/ethyl acetate mixture (96:4), cholesteryl phenyl ether **12f** (31 mg, 22%) was eluted. Further elution with hexane/ethyl acetate (93:7) afforded 3β-*O*-(1’,2’:3’,4’-di-*O*-isopropylidene-α-D-galactopyranos-6’-yl)-cholest-5-ene (**11**, 108 mg, 58%), followed by cholesterol **1** (5 mg, 4%) eluted with hexane/ethyl acetate (9:1).

Glycosylation product **11** was described in our previous paper [4]. Also, other products of the electrochemical reactions (compounds **2**, **13**, **14**, and **15**) were described in our previous papers [1–5]. The isomerization products, i.e., 3β-cholesteryl ethers **12b** [13], **12c** [14], **12e** [15], **12f** [14], **12g** [5], and **12h** [16], are known compounds, except for **12d** which was obtained during electrochemical reaction of 3α,5α-cyclocholestan-6β-yl isopropyl ether (**6d**). See the Supporting Information File 1 for full experimental data.

## Supporting Information

File 1 Experimental section including ^1^H, ^13^C NMR, and mass spectra for all new compounds.
