# Supplementary material for: 3α,5α-Cyclocholestan-6β-yl ethers as donors of the cholesterol moiety for the electrochemical synthesis of cholesterol glycoconjugates
Source: Beilstein J Org Chem. 2015 Jan 26;11:162–8. doi: 10.3762/bjoc.11.16 (PMC4362039; doi:10.3762/bjoc.11.16)
Supplement: File 1 — Experimental section including 1H, 13C NMR, and mass spectra for all new compounds. [file Beilstein_J_Org_Chem-11-162-s001.pdf]

Supporting Information

for

3α,5α-Cyclocholestan-6β-yl ethers as donors of the cholesterol moiety for the electrochemical synthesis of cholesterol glycoconjugates

Aneta M. Tomkiel[1], Adam Biedrzycki[1], Jolanta Płoszyńska[2], Dorota Naróg[2], Andrzej Sobkowiak[2] and Jacek W. Morzycki*[1]

Address: [1]Institute of Chemistry, University of Białystok, ul. Ciołkowskiego 1K, 15-245 Białystok, Poland and [2]Faculty of Chemistry, Rzeszów University of Technology, P.O. Box 85, 35-959 Rzeszów, Poland

Email: Jacek W. Morzycki – morzycki@uwb.edu.pl

* Corresponding author

Experimental section including ^{1}H, ^{13}C NMR, and mass spectra for all new compounds

Experimental

Cyclic voltammograms were recorded with iR compensation at 25 °C using a three-electrode potentiostat (Princeton Applied Research, model Parstat 2273). The experiments were conducted in a 3-mL electrochemical cell with an argon-purge system. The working electrode was a Bioanalytical Systems platinum inlay (1 mm in diameter), the auxiliary electrode was a platinum mesh (contained in a glass tube with a medium porosity glassfrit), and the reference electrode was Ag/0.1 M $AgNO_3$ in acetonitrile. The latter was contained in a Pyrex tube with a cracked softglass tip which was placed inside a Luggin capillary. Before each experiment, the working electrode was polished using Buehler Micropolish Alumina Gamma 3B and a Buehler Microcloth polishing cloth, rinsed with dichloromethane and dried. In all of the measurements, 0.2M solution of tetrabutylammonium tetrafluoroborate ($TBABF_4$) from Aldrich in dichloromethane was used as a supporting electrolyte.

The preparative electrolyses were performed with a potentiostat/galvanostat (Princeton Applied Research, model Parstat 2273) under galvanostatic conditions using a current that was equal in a typical experiment to 7.5 mA and a reaction time of 4000 s. The current applied was the maximum current available for the electrolysis set-up being used (power supply and ohmic resistance). The reactions were monitored by TLC and stopped when no further increase in the concentration of the glycosylation products was observed. A divided H-cell was used in which the cathodic and anodic compartments (3.5 mL of electrolyte each) were separated by a glass frit. In all measurements, 0.1 M solution of tetrabutylammonium tetrafluoroborate ($TBABF_4$) from Aldrich in dichloromethane was used as a supporting electrolyte. The steroid (0.30 mmol) and sugar (0.36 mmol) substrates were introduced into the anodic compartment together with 0.3 g of 3 Å molecular sieves added to eliminate traces of water, whereas anionite (1.5–2 g, Dowex 2 × 8, 200–400 mesh, perchlorate form) was placed in the cathodic compartment to eliminate chloride ions that are formed by the reduction of dichloromethane. The solutions in both compartments were stirred during electrolysis and, additionally, a continuous flow of argon was applied in the anodic compartment. A platinum mesh was used as a cathode and a platinum plate (2 × 1.5 cm) was used as an anode. All measurements were performed at 25 °C.

The sugar (1,2:3,4-di-*O*-isopropylidene-α-D-galactopyranose; **7**) [1] and steroidal substrates, 3α,5α-cyclocholestan-6β-ol (*i*-cholesterol; **6a**) [2], 6β-methoxy-3α,5α-cyclocholestane (**6b**) [3], and 6β-ethoxy-3α,5α-cyclocholestane (**6c**) [4], were prepared according to known procedures.

Melting points were determined on a Toledo Mettler-MP70 apparatus. ^{1}H and ^{13}C NMR (400 and 100 MHz, respectively) spectra were recorded on a Bruker Avance II spectrometer in $CDCl_3$ solutions with TMS as the internal standard (only selected signals in the ^{1}H NMR spectra are reported; sugar protons are marked with the 'prime' index). Infrared spectra were recorded on a Nicolet series II Magna-IR 550 FTIR spectrometer in chloroform solutions. Mass spectra were recorded at 70 eV with a time-of-flight (TOF) AMD-604 spectrometer with electrospray ionization (ESI) or AutoSpec Premier (Waters) (EI).

Merck Silica Gel 60, F 256 TLC aluminum sheets were applied for thin-layer chromatographic analysis. For a visualization of the products, a 5% solution of phosphomolybdic acid in ethanol was used. The reaction products were separated by column chromatography performed on a 70–230 mesh silica gel (J. T. Baker).

Synthesis of 6β-benzyloxy-3α,5α-cyclocholestane (**6e**)

To cholesteryl *p*-tosylate (1 g; 1.9 mmol) dissolved in dioxane (50 mL) freshly dried potassium acetate (0.8 g; 5.7 mmol; 3 equiv.) and benzyl alcohol (6.2 g; 57 mmol; 30 equiv.) were added. The reaction mixture was refluxed for 24 h. After cooling it was poured into water (200 mL) and extracted with benzene (3 × 100 mL). The extract was dried over anhydrous Na_2SO_4 and evaporated to dryness *in vacuo*. The residue was subjected to silica gel column chromatography. Elution with CH_2Cl_2/hexane (1:9) afforded 6β-benzyloxy-3α,5α-cyclocholestane (**6e**; 0.43 g; 48%).

6e: Colorless oil; $[\alpha]_D^{20}$ +37.0 (*c* 1.0, $CHCl_3$); Rf = 0.31 (hexane-AcOEt 98:2); IR, ν_{max} (cm^{-1}): 3064, 1496, 1093, 1067; ^{1}H NMR (ppm), δ: 7.35 (m, 4H, H-Ar), 7.27 (m, 1H, H-Ar), 4.66 (d, 1H, *J* = 12.5 Hz, O-CH_2Ph), 4.50 (d, 1H, *J* = 12.5 Hz, O-CH_2Ph), 2.98 (m, 1H, H-6), 1.11 (s, 3H, H-19), 0.93 (d, 3H, *J* = 6.5 Hz, H-21), 0.887 (d, 3H, *J* = 6.6 Hz, H-26 or H-27), 0.883 (d, 3H, *J* = 6.6 Hz, H-26 or H-27), 0.72 (s, 3H, H-18), 0.67 (dd, 1H, *J* = 4.9 Hz, *J* = 4.1 Hz, H-4α), 0.41 (dd, 1H, *J* = 8.0 Hz, *J* = 4.9 Hz, H-4β); ^{13}C NMR (ppm), δ: 140.1 (C), 128.2 (CH), 127.1 (CH), 126.9 (CH), 80.2 (CH), 70.0 (CH_2), 56.6 (CH), 56.4 (CH), 48.1 (CH), 43.3 (C), 42.8 (C), 40.4 (CH_2), 39.5 (CH_2), 36.2 (CH_2), 35.8 (CH), 35.7 (C), 35.4 (CH_2), 33.4 (CH_2), 30.6 (CH), 28.3 (CH_2), 28.0 (CH), 25.0 (CH_2), 24.2 (CH_2), 23.9 (CH_2), 22.84 (CH_2), 22.81 (CH_3), 22.6 (CH_3), 21.8 (CH), 19.5 (CH_3), 18.7 (CH_3), 13.2 (CH_2), 12.3 (CH_3); EI MS, *m/z:* 476 (M^+, 35%), 461 [$(M-Me)^+$, 7%], 385 [$(M-Bn)^+$, 77%], 370 [$(M-Bn-Me)^+$, 67%], 91 (100%).

6β-Isopropyloxy-3α,5α-cyclocholestane (**6d**) was obtained in a similar way (compound **6d** was eluted with hexane).

6d: Colorless oil; $[\alpha]_D^{20}$ +43.4 (*c* 1.0, $CHCl_3$); Rf = 0.39 (hexane-AcOEt 98:2); IR, ν_{max} (cm^{-1}): 1138, 1124, 1041; ^{1}H NMR (ppm), δ: 3.74 (h, 1H, *J* = 6.1 Hz, O-$CH(Me)_2$), 2.93 (m, 1H, H-6), 1.08 (d, 6H, *J* = 6.2 Hz, $(CH_3)_2CH$), 1.02 (s, 3H, H-19), 0.93 (d, 3H, *J* = 6.6 Hz, H-21), 0.881 (d, 3H, *J* = 6.6 Hz, H-26 or H-27), 0.877 (d, 3H, *J* = 6.6 Hz, H-26 or H-27), 0.73 (s, 3H, H-18), 0.58 (dd, 1H, *J* = 4.9 Hz, *J* = 3.9 Hz, H-4α), 0.33 (dd, 1H, *J* = 7.9 Hz, *J* = 4.9 Hz, H-4β); ^{13}C NMR (ppm), δ: 76.9 (CH), 67.1 (CH), 56.6 (CH), 56.4 (CH), 48.2 (CH), 43.2 (C), 42.8 (C), 40.4 (CH_2), 39.6 (CH_2), 36.4 (C), 36.2 (CH_2), 35.9 (CH), 35.8 (CH_2), 33.4 (CH_2), 30.4 (CH), 28.4 (CH_2), 28.0 (CH), 25.1 (CH_2), 24.3 (CH_2), 23.9 (CH_2), 22.9 (CH_2), 22.8 (CH_3), 22.6 (CH_3), 22.52 (CH_3), 22.47 (CH_3), 22.3 (CH), 19.6 (CH_3), 18.7 (CH_3), 12.8 (CH_2), 12.2 (CH_3); EI MS, *m/z:* 428 [M^+, 56%], 413 [$(M-Me)^+$, 20%], 386 [$(M-propene)^+$, 17%], 371 [$(M-Me-propene)^+$, 81%], 43 (100%).

6β-(4-Hydroxyphenyloxy)-3α,5α-cyclocholestane (**6g**) was obtained in a similar way (compound **6g** was eluted with a hexane-ethyl acetate (93:7) mixture).

6g: Beige crystalline material, mp 54-56 °C (CH_2Cl_2-hexane); $[\alpha]_D^{20}$ +24.4 (*c* 0.33, $CHCl_3$); Rf = 0.27 (hexane-AcOEt 9:1); IR, ν_{max}(cm^{-1}): 3601, 3340, 1601, 1507, 1175, 827; ^{1}H NMR (ppm), *δ*: 6.79 (d, 2H, *J* = 9.0 Hz, H-Ar), 6.72 (d, 2H, *J* = 9.0 Hz, H-Ar), 4.36 (s, 1H, -OH), 3.64 (m, 1H, H-6), 1.16 (s, 3H, H-19), 0.93 (d, 3H, *J* = 6.5 Hz, H-21), 0.876 (d, 3H, *J* = 6.6 Hz, H-26 or H-27), 0.872 (d, 3H, *J* = 6.6 Hz, H-26 or H-27), 0.73 (s, 3H, H-18), 0.60 (dd, 1H, *J* = 5.1 Hz, *J* = 3.9 Hz, H-4α), 0.34 (dd, 1H, *J* = 7.9 Hz, *J* = 5.1 Hz, H-4β); ^{13}C NMR (ppm), *δ*: 152.4 (C), 150.1 (C), 119.2 (CH), 116.0 (CH), 82.5 (CH), 56.39 (CH), 56.35 (CH), 47.7 (CH), 43.2 (C), 42.7 (C), 40.2 (CH_2), 39.5 (CH_2), 36.6 (C), 36.2 (CH_2), 35.8 (CH), 34.5 (CH_2), 33.4 (CH_2), 30.3 (CH), 28.2 (CH_2), 28.0 (CH), 25.0 (CH_2), 24.1 (CH_2), 23.8 (CH_2), 23.5 (CH), 22.8 (CH_3), 22.7 (CH_2), 22.5 (CH_3), 19.9 (CH_3), 18.7 (CH_3), 12.6 (CH_2), 12.1 (CH_3); EI MS, *m/z:* 478 (M^+, 1%), 369 [$(M$-*p*-$OH-PhO)^+$, 100%].

Synthesis of 6β-phenyloxy-3α,5α-cyclocholestane (**6f**)

Phenol (10 g; 0.1 mol, 36 equiv.) was melted at 42 °C. Then cholesteryl *p*-tosylate (1.5 g; 2.8 mmol) and freshly dried potassium acetate (0.8 g; 8.2 mmol, 3 equiv.) were added. The stirred reaction mixture was maintained at this temperature for 2 h. After cooling it was poured into 100 mL of 1 M NaOH solution and extracted with benzene (3 × 100 mL). The extract was dried over anhydrous Na_2SO_4 and evaporated to dryness *in vacuo*. The reaction products were separated by silica gel column chromatography. 6β-Phenyloxy-3α,5α-cyclocholestane (**6f**; 0.27 g, 21%) was eluted with hexane. Further elution with a hexane/ethyl acetate (99:1) mixture afforded cholesteryl phenyl ether **12f** (0.41 g; 32%).

6f: White crystals, mp 58-61 °C (AcOEt-hexane); $[\alpha]_D^{20}$ +36.5 (*c* 0.33, $CHCl_3$); Rf = 0.28 (hexane); IR, ν_{max} (cm^{-1}): 3064, 1597, 1243; ^1H NMR (ppm), δ: 7.26 (m, 2H, H-Ar), 6.91 (m, 3H, H-Ar), 3.82 (m, 1H, H-6), 1.19 (s, 3H, H-19), 0.94 (d, 3H, *J* = 6.5 Hz, H-21), 0.893 (d, 3H, *J* = 6.6 Hz, H-26 or H-27), 0.889 (d, 3H, *J* = 6.6 Hz, H-26 or H-27), 0.74 (s, 3H, H-18), 0.62 (dd, 1H, *J* = 5.2 Hz, *J* = 3.9 Hz, H-4α), 0.36 (dd, 1H, *J* = 7.9 Hz, *J* = 5.2 Hz, H-4β); ^{13}C NMR (ppm), *δ*: 158.6 (C), 129.2 (CH), 120.5 (CH), 116.9 (CH), 80.7 (CH), 56.4 (CH), 56.3 (CH), 47.7 (CH), 43.2 (C), 42.7 (C), 40.2 (CH_2), 39.5 (CH_2), 36.7 (C), 36.2 (CH_2), 35.8 (CH), 34.3 (CH_2), 33.3 (CH_2), 30.4 (CH), 28.2 (CH_2), 28.0 (CH), 25.1 (CH_2), 24.1 (CH_2), 23.9 (CH_2), 23.6 (CH), 22.81 (CH_3), 22.76 (CH_2), 22.6 (CH_3), 19.8 (CH_3), 18.7 (CH_3), 12.6 (CH_2), 12.2 (CH_3); EI MS, *m/z:* 462 (M^+, 1%), 369 [$(M\text{-}PhO)^+$, 100%].

Synthesis of 6β-*t*-butyldimethylsilyloxy-3α,5α-cyclocholestane (**6h**)

i-Cholesterol (1 g; 2.6 mmola), TBDMSCl (584 mg; 3.88 mmol, 1.5 equiv.), imidazole (530 mg; 7.8 mmol; 3 equiv.), and DMAP (64 mg; 0.52 mmol; 0.2 equiv.) were dissolved in 16 mL of anhydrous DMF. The reaction was carried out for 5 h (TLC control) at 80 °C under argon. The reaction was poured into water and extracted with benzene. The extract was dried with anhydrous Na_2SO_4 and the solvent was evaporated *in vacuo*. The residue was subjected to flash column chromatography with hexane as an eluent. Yield of 6β-*t*-butyldimethylsilyloxy-3α,5α-cyclocholestane (**6h**) - 688 mg (53%).

6h: White crystals, mp 70-71 °C (AcOEt-hexane); $[\alpha]_D^{20}$ +27.0 (*c* 1.0, $CHCl_3$); Rf = 0.84 (hexane); IR, ν_{max} (cm^{-1}): 1254, 1065; ^1H NMR (ppm), *δ*: 3.20 (m, 1H, H-6), 1.02 (s, 3H, H-19), 0.93 (d, 3H, *J* = 6.5 Hz, H-21), 0.89 (s, 9H, *t*-Bu), 0.881 (d, 3H, *J* = 6.6 Hz, H-26 or H-27), 0.877 (d, 3H, *J* = 6.6 Hz, H-26 or H-27), 0.72 (s, 3H, H-18), 0.48 (dd, 1H, *J* = 4.9 Hz, *J* = 3.8 Hz, H-4α), 0.18 (dd, 1H, *J* = 7.9 Hz, *J* = 4.9 Hz, H-4β), 0.01 (s, 3H, Si-CH_3), -0.01 (s, 3H, Si-CH_3); ^{13}C NMR (ppm), *δ*: 74.4 (CH), 56.4 (CH), 56.3 (CH), 47.9 (CH), 43.3 (C), 42.7 (C), 40.4 (CH_2), 39.6 (CH_2), 39.1 (CH_2), 38.3 (C), 36.3 (CH_2), 35.9 (CH), 33.4 (CH_2), 30.0 (CH), 28.4 (CH_2), 28.1 (CH), 25.8 (CH_3), 25.2 (CH_2), 24.3 (CH_2), 23.9 (CH_2), 23.6 (CH), 22.9 (CH_3), 22.8 (CH_2), 22.6 (CH_3), 20.2 (CH_3), 18.8 (CH_3), 18.0 (C), 12.8 (CH_2), 12.0 (CH_3), -4.5 (CH_3), -4.8 (CH_3); ESI MS, *m/z:* 523 [$(M+Na)^+$, 20%].

Typical electrochemical experiment. Anodic oxidation of 6β-phenyloxy-3α,5α-cyclocholestane (**6f**) in the presence of 1,2:3,4-di-*O*-isopropylidene-D-galactopyranose (**7**)

6β-Phenyloxy-3α,5α-cyclocholestane (138 mg; 0.30 mmol) and 1,2:3,4-di-*O*-isopropylidene-D-galactopyranose (94 mg; 0.36 mmol) were dissolved in a 0.1 M solution of tetrabutylammonium-tetrafluoroborate in dichloromethane (3.5 mL) and introduced into the anodic compartment together with 0.5 g 3 Å molecular sieves to eliminate traces of water. The same supporting electrolyte was placed in the cathodic compartment with anionite (2 g, Dowex 2x8, 200–400 mesh,

perchlorate form) added. Preparative electrolysis was carried out in a divided H-cell in which the cathodic and anodic compartments (3.5 mL of electrolytes each) were separated by a glass frit under galvanostatic conditions. A direct current 7.5 mA was run for 4000 s. A platinum mesh was used as a cathode and a platinum plate (2 × 1.5 cm) was used as an anode. Ag/0.1 M $AgNO_3$ in acetonitrile electrode was used as a reference. When the electrolysis was completed, the solvent was removed from the reaction mixture and the products were separated by silica gel column chromatography. The hexane elution afforded diene **13** (1 mg; 1%) and cholesteryl chloride **14** (1 mg; 1%). With the hexane-ethyl acetate mixture (96:4), cholesteryl phenyl ether **12f** (31 mg; 22%) was eluted. Further elution with hexane-ethyl acetate (93:7) afforded 3β-*O*-(1′,2′:3′,4′-di-*O*-isopropylidene-α-D-galactopyranos-6′-yl)-cholest-5-ene **11** (108 mg; 58%), followed by cholesterol **1** (5 mg, 4%) eluted with hexane/ethyl acetate (9:1).

Glycosylation product **11** was described in our previous report [5]. Also, other products of the electrochemical reactions (compounds **2**, **13**, **14**, and **15**) were described in our previous papers [6-9]. The isomerization products, i.e., 3β-cholesteryl ethers **12b** [10], **12c** [11], **12e** [12], **12f** [12], **12g** [9],and **12h** [13], are known compounds, except for **12d** which was obtained during electrochemical reaction of 3α,5α-cyclocholestan-6β-yl isopropyl ether (**6d**).

12d: White crystals, mp 122-124 °C (AcOEt-hexane); $[\alpha]_D^{20}$ -24.1 (*c* 1.0, $CHCl_3$); Rf = 0.44 (hexane-AcOEt 95:5); IR, ν_{max} (cm^{-1}): 1125, 1064; ^1H NMR (ppm), δ: 5.35 (m, 1H, H-6), 3.73 (h, 1H, *J* = 6.1 Hz, O-CH$(Me)_2$), 3.22 (m, 1H, H-6) 1.151 (d, 3H, *J* = 6.1 Hz, CH_3-isopropyl), 1.148 (d, 3H, *J* = 6.1 Hz, CH_3-isopropyl), 1.01 (s, 3H, H-19), 0.93 (d, 3H, *J* = 6.6 Hz, H-21), 0.877 (d, 3H, *J* = 6.6 Hz, H-26 or H-27), 0.873 (d, 3H, *J* = 6.6 Hz, H-26 or H-27), 0.67 (s, 3H, H-18); ^{13}C NMR (ppm), δ: 141.4 (C), 121.3 (CH), 76.3 (CH), 68.5 (CH), 56.8 (CH), 56.2 (CH), 50.3 (CH), 42.3 (C), 40.0 (CH_2), 39.8 (CH_2), 39.5 (CH_2), 37.5 (CH_2), 36.9 (C), 36.2 (CH_2), 35.8 (CH), 32.0 (CH_2), 31.9 (CH), 29.3 (CH_2), 28.2 (CH_2), 28.0 (CH), 24.3 (CH_2), 23.8 (CH_2), 23.03 (CH_3), 22.95 (CH_3), 22.8 (CH_3), 22.6 (CH_3), 21.1 (CH_2), 19.4 (CH_3), 18.7 (CH_3), 11.9 (CH_3); EI MS, *m/z:* 428 [M^+, 15%], 370 [$(M$-*i*-Pr-Me$)^+$, 63%], 329 (100%).

References:

1. Calinaud, P.; Gelas, J. Synthesis of isopropylidene, benzylidene, and related acetals. In *Preparative Carbohydrate Chemistry*; Hanessian, S., Ed.; Marcel Dekker: New York, USA, 1997; pp 3–34.
2. Patel, M. S.; Peal, W. J. *J. Chem. Soc.* **1963**, 1544–1546.
3. Ford, E. G.; Wallis, E. S. *J. Am. Chem. Soc.* **1937**, *59*, 1415-1416.
4. Beynon, J. H.; Heilbron, I. M.; Spring, F. S. *J. Chem. Soc*. **1936**, 907-910.
5. Tomkiel, A. M.; Brzezinski, K.; Łotowski, Z.; Siergiejczyk, L.; Wałejko, P.; Witkowski, S.; Kowalski, J.; Płoszyńska, J.;Sobkowiak, A.; Morzycki, J. W. *Tetrahedron* **2013**, *69*, 8904–8913.
6. Kowalski, J.; Morzycki, J. W.; Sobkowiak, A.; Wilczewska, A. Z. *Steroids* **2008**, *73*, 543–548.
7. Kowalski, J.; Płoszyńska, J.; Sobkowiak, A.; Morzycki, J. W.; Wilczewska, A. Z. *J. Electroanal. Chem.* **2005**, *585*, 275–280.
8. Morzycki, J. W.; Łotowski, Z.; Siergiejczyk, L.; Wałejko, P.; Witkowski, S.; Kowalski, J.; Płoszyńska, J.; Sobkowiak, A. *Carbohydr. Res.* **2010**, *345*, 1051–1055.
9. Tomkiel, A. M.; Kowalski, J.; Płoszyńska, J.; Siergiejczyk, L.; Łotowski, Z.; Sobkowiak, A.; Morzycki, J. W. *Steroids* **2014**, *82*, 60–67.
10. Narayanan, C. R.; Iyer, K. N. *J. Org. Chem*. **1965**, *30*, 1734–1736.
11. Wang, B.; Du, H.; Zhang, J. *Steroids* **2011**, *76*, 204–209.
12. Lu, B.; Li, L. J.; Li, T. S.; Li, J. T. *J Chem Res (S)* **1998**, 604–605.
13. Bartoszewicz, A.; Kalek, M.; Stawinski, J. *Tetrahedron* **2008**, *64*, 8843–8850.

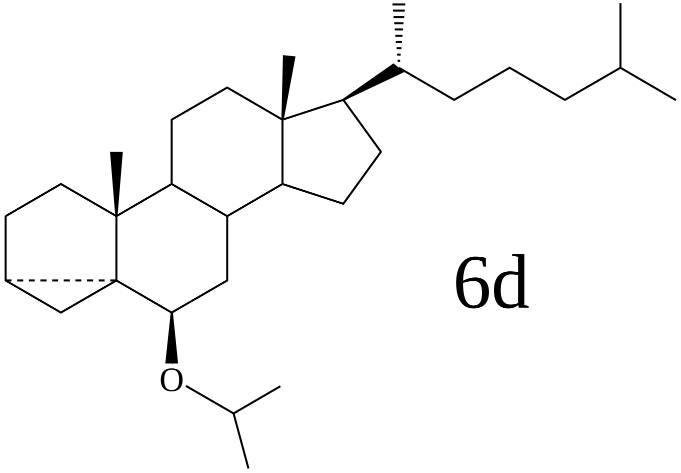

6d

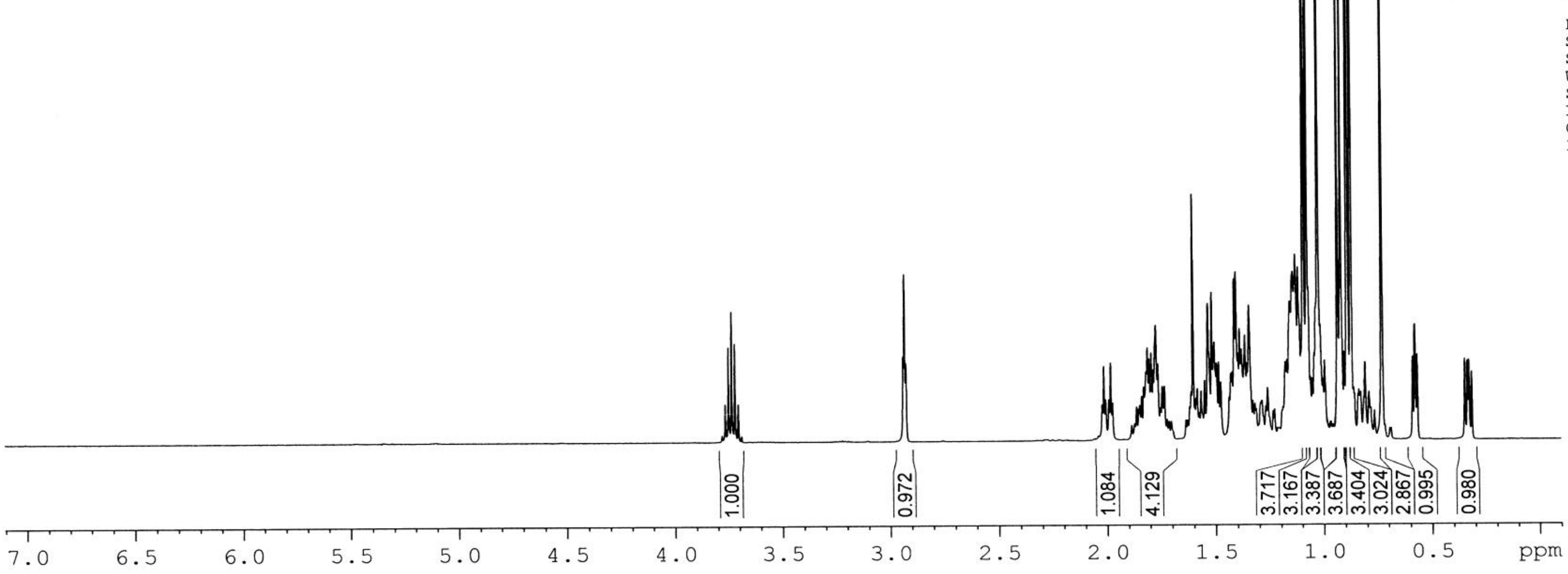


```
Current Data Parameters
NAME                 AT 214
EXPNO                     1
PROCNO                    1

F2 - Acquisition Parameters
Date_              20131014
Time                  14.53
INSTRUM               spect
PROBHD   5 mm PABBO BB-
PULPROG                zg30
TD                    65536
SOLVENT               CDCl3
NS                       64
DS                        0
SWH             8223.685 Hz
FIDRES          0.125483 Hz
AQ            3.9846387 sec
RG                     50.8
DW               60.800 usec
DE                 8.00 usec
TE                  673.2 K
D1           1.00000000 sec
TD0                       1

======== CHANNEL f1 ========
NUC1                     1H
P1                11.15 usec
PL1               -3.00 dB
SFO1        400.1524711 MHz

F2 - Processing parameters
SI                    32768
SF          400.1500000 MHz
WDW                      GM
SSB                       0
LB                 -0.20 Hz
GB                      0.2
PC                     1.00
```

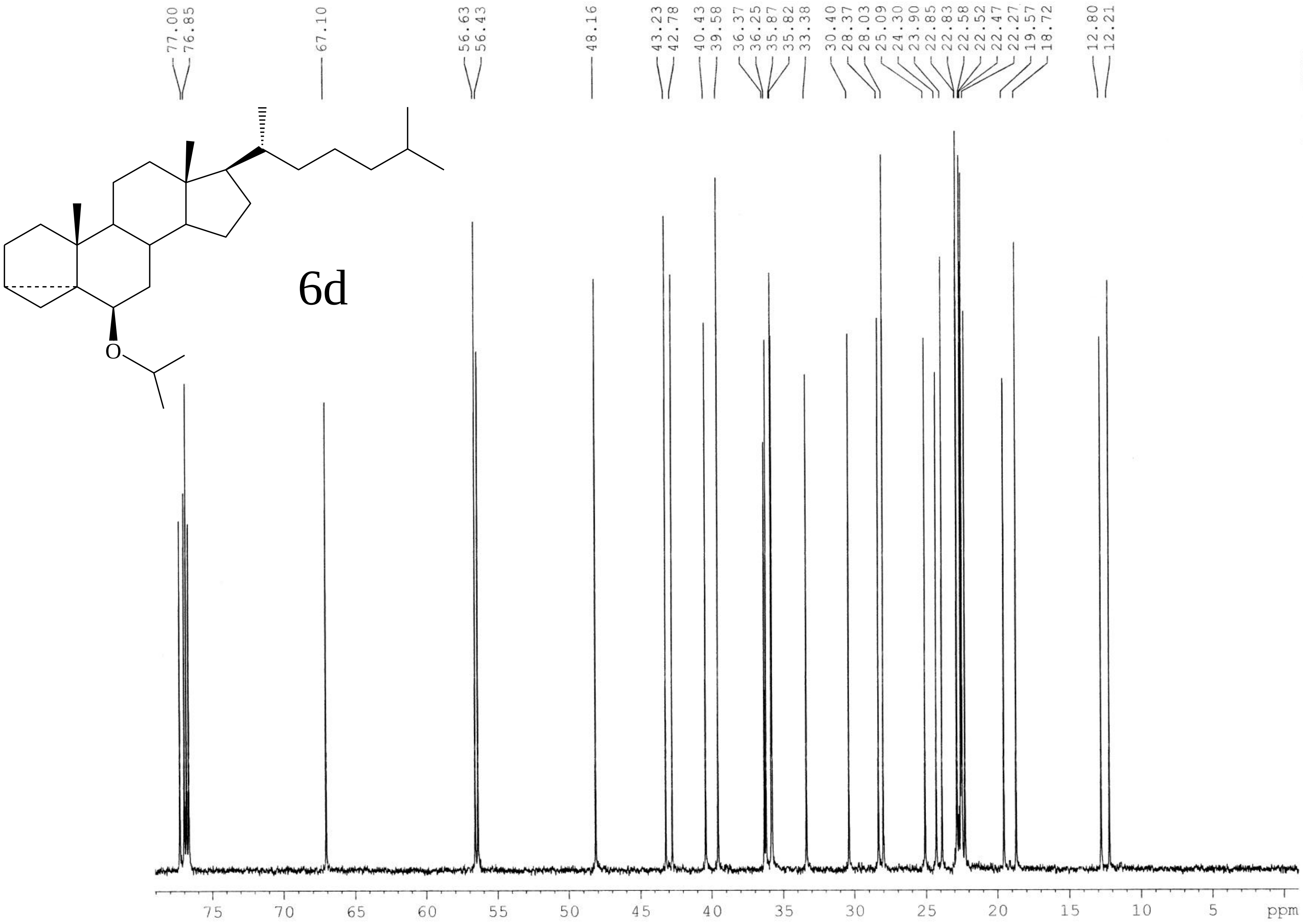


```
Current Data Parameters
NAME              AT 216f1
EXPNO                    2
PROCNO                   1

F2 - Acquisition Parameters
Date_             20131122
Time                 17.40
INSTRUM              spect
PROBHD    5 mm PABBO BB-
PULPROG            zgpg30
TD                   65536
SOLVENT              CDCl3
NS                     224
DS                       4
SWH              27173.912 Hz
FIDRES            0.414641 Hz
AQ               1.2059124 sec
RG                    1030
DW                  18.400 usec
DE                    6.00 usec
TE                   673.2 K
D1              2.00000000 sec
d11             0.03000000 sec
DELTA           1.89999998 sec
TD0                      1

======== CHANNEL f1 ========
NUC1                   13C
P1                   12.25 usec
PL1                  -1.00 dB
SFO1           100.6288660 MHz

======== CHANNEL f2 ========
CPDPRG2            waltz16
NUC2                    1H
PCPD2               100.00 usec
PL2                  -3.00 dB
PL12                 13.65 dB
PL13                 18.00 dB
SFO2           400.1516006 MHz

F2 - Processing parameters
SI                   32768
SF             100.6177963 MHz
WDW                     EM
SSB                      0
LB                    1.00 Hz
GB                       0
PC                    0.20
```

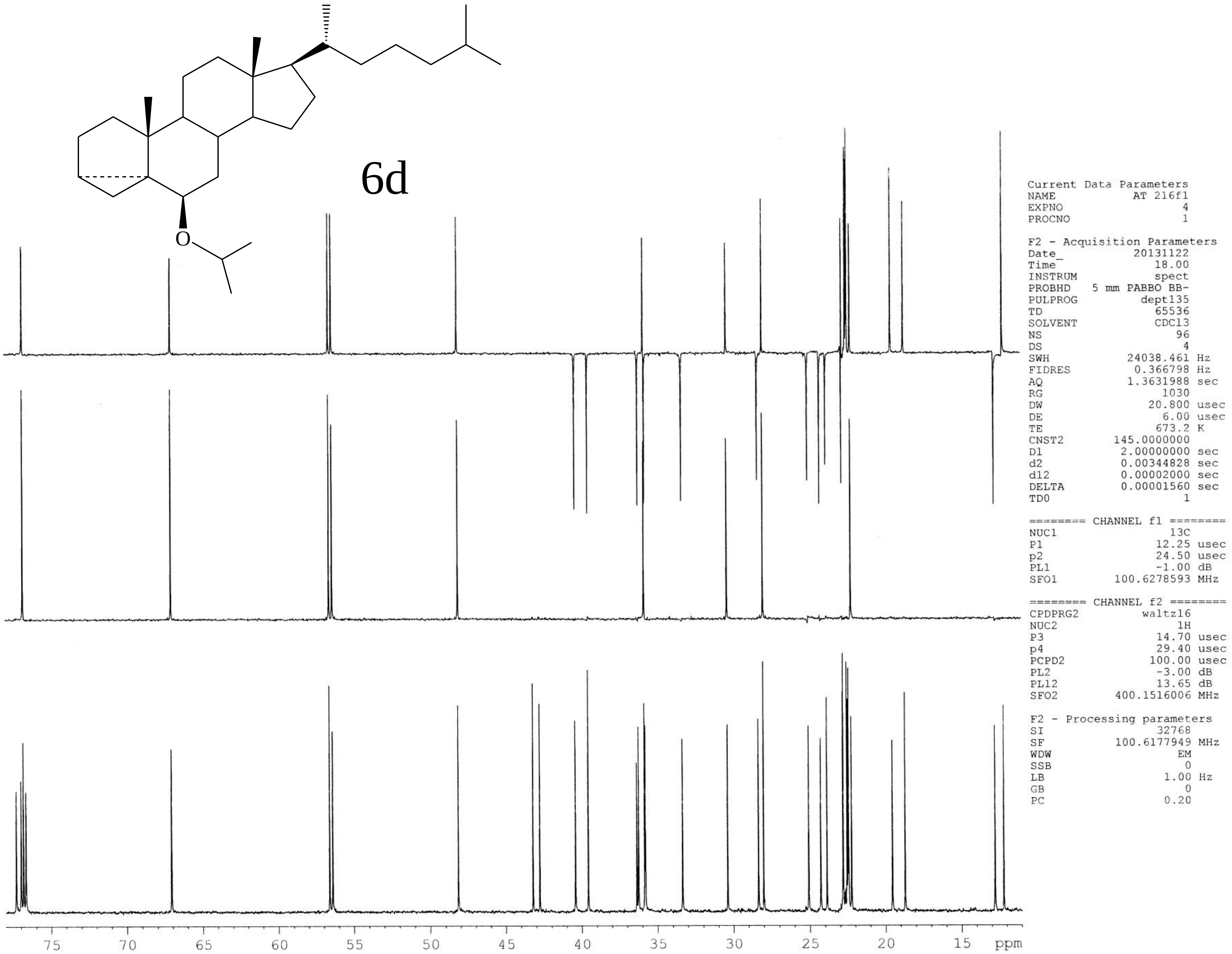
6d
O
Current Data Parameters
NAME AT 216f1
EXPNO 4
PROCNO 1
F2 - Acquisition Parameters
Date_ 20131122
Time 18.00
INSTRUM spect
PROBHD 5 mm PABBO BB-
PULPROG dept135
TD 65536
SOLVENT CDCl3
NS 96
DS 4
SWH 24038.461 Hz
FIDRES 0.366798 Hz
AQ 1.3631988 sec
RG 1030
DW 20.800 usec
DE 6.00 usec
TE 673.2 K
CNST2 145.0000000
D1 2.00000000 sec
d2 0.00344828 sec
d12 0.00002000 sec
DELTA 0.00001560 sec
TD0 1
======== CHANNEL f1 ========
NUC1 13C
P1 12.25 usec
p2 24.50 usec
PL1 -1.00 dB
SFO1 100.6278593 MHz
======== CHANNEL f2 ========
CPDPRG2 waltz16
NUC2 1H
P3 14.70 usec
p4 29.40 usec
PCPD2 100.00 usec
PL2 -3.00 dB
PL12 13.65 dB
SFO2 400.1516006 MHz
F2 - Processing parameters
SI 32768
SF 100.6177949 MHz
WDW EM
SSB 0
LB 1.00 Hz
GB 0
PC 0.20
75 70 65 60 55 50 45 40 35 30 25 20 15 ppm

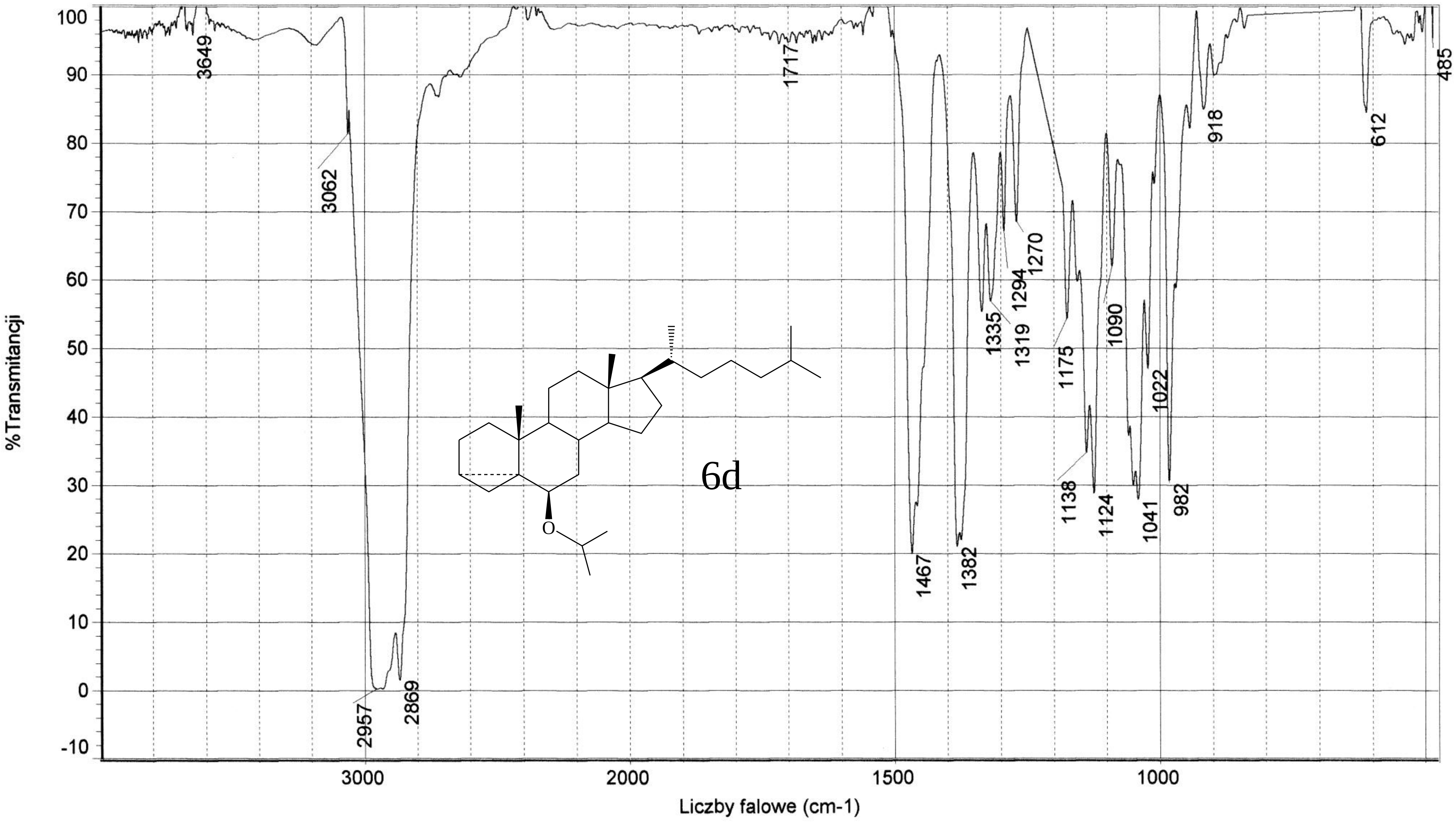
%Transmitancji
Liczby falowe (cm-1)
3649
3062
2957
2869
1717
1467
1382
1335
1319
1294
1270
1175
1138
1124
1090
1041
1022
982
918
612
485
O

6d

AUTOSPEC

AT214

ub_jm3232 12 (0.952) Cm (9:17)

22-Oct-2013 10:01:23

Magnet EI+
3.58e3

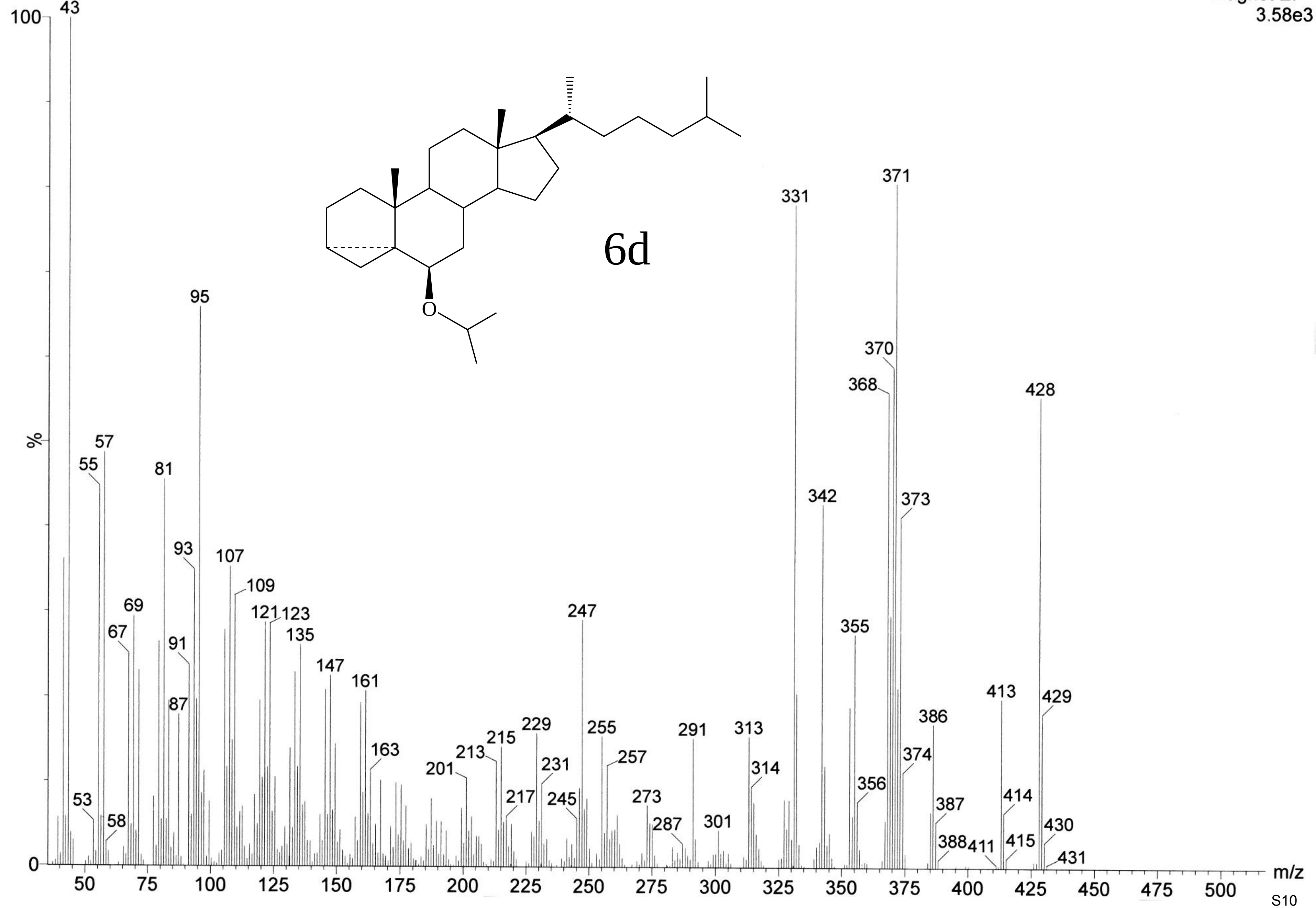

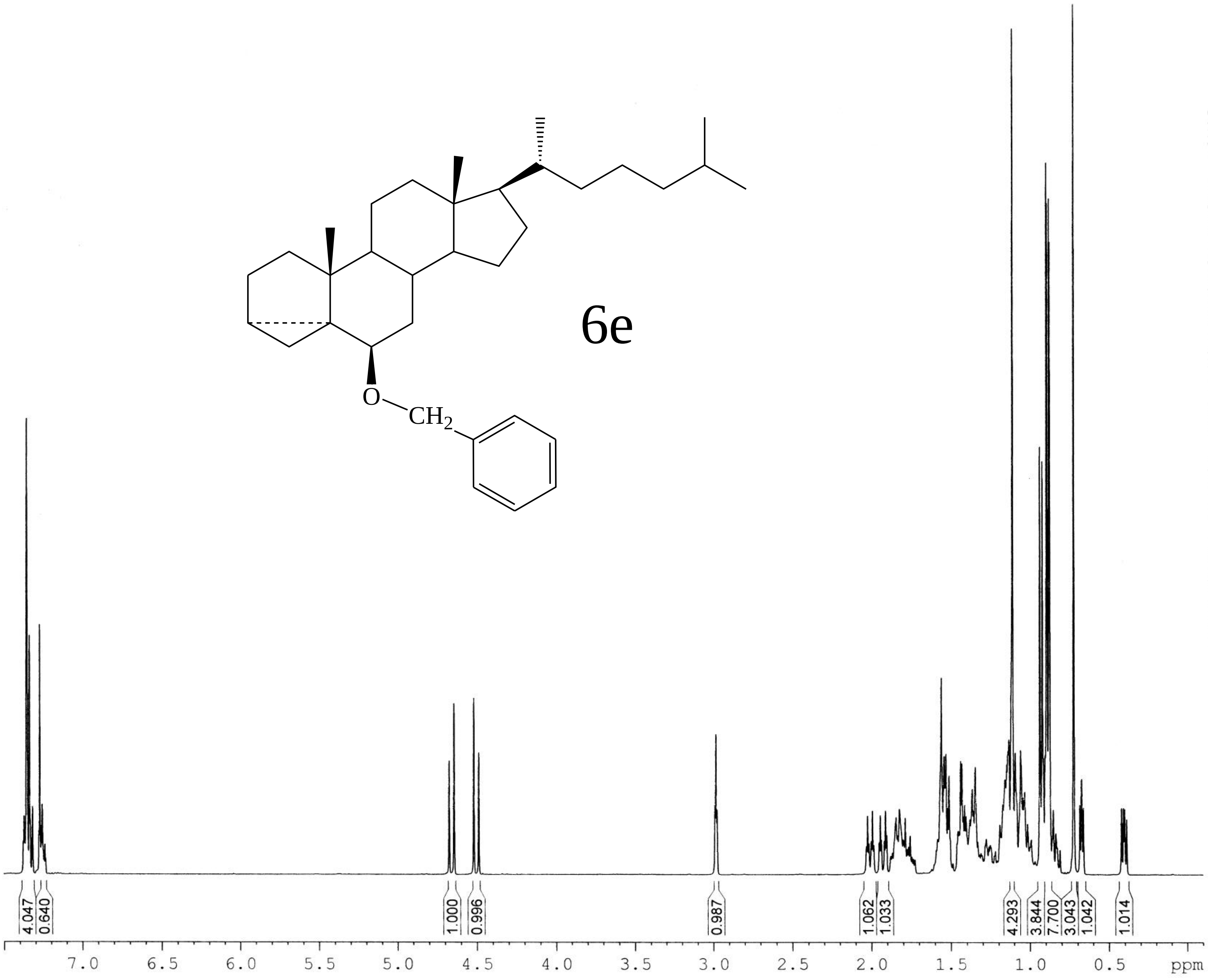

Current Data Parameters
NAME AT benzylowy W6f1
EXPNO 1
PROCNO 1

F2 - Acquisition Parameters
Date_ 20131129
Time 14.28
INSTRUM spect
PROBHD 5 mm PABBO BB-
PULPROG zg30
TD 65536
SOLVENT CDCl3
NS 128
DS 0
SWH 8223.685 Hz
FIDRES 0.125483 Hz
AQ 3.9846387 sec
RG 114
DW 60.800 usec
DE 8.00 usec
TE 673.2 K
D1 1.00000000 sec
TD0 1

======== CHANNEL f1 ========
NUC1 1H
P1 11.15 usec
PL1 -3.00 dB
SFO1 400.1524711 MHz

F2 - Processing parameters
SI 32768
SF 400.1500000 MHz
WDW GM
SSB 0
LB -0.20 Hz
GB 0.2
PC 1.00

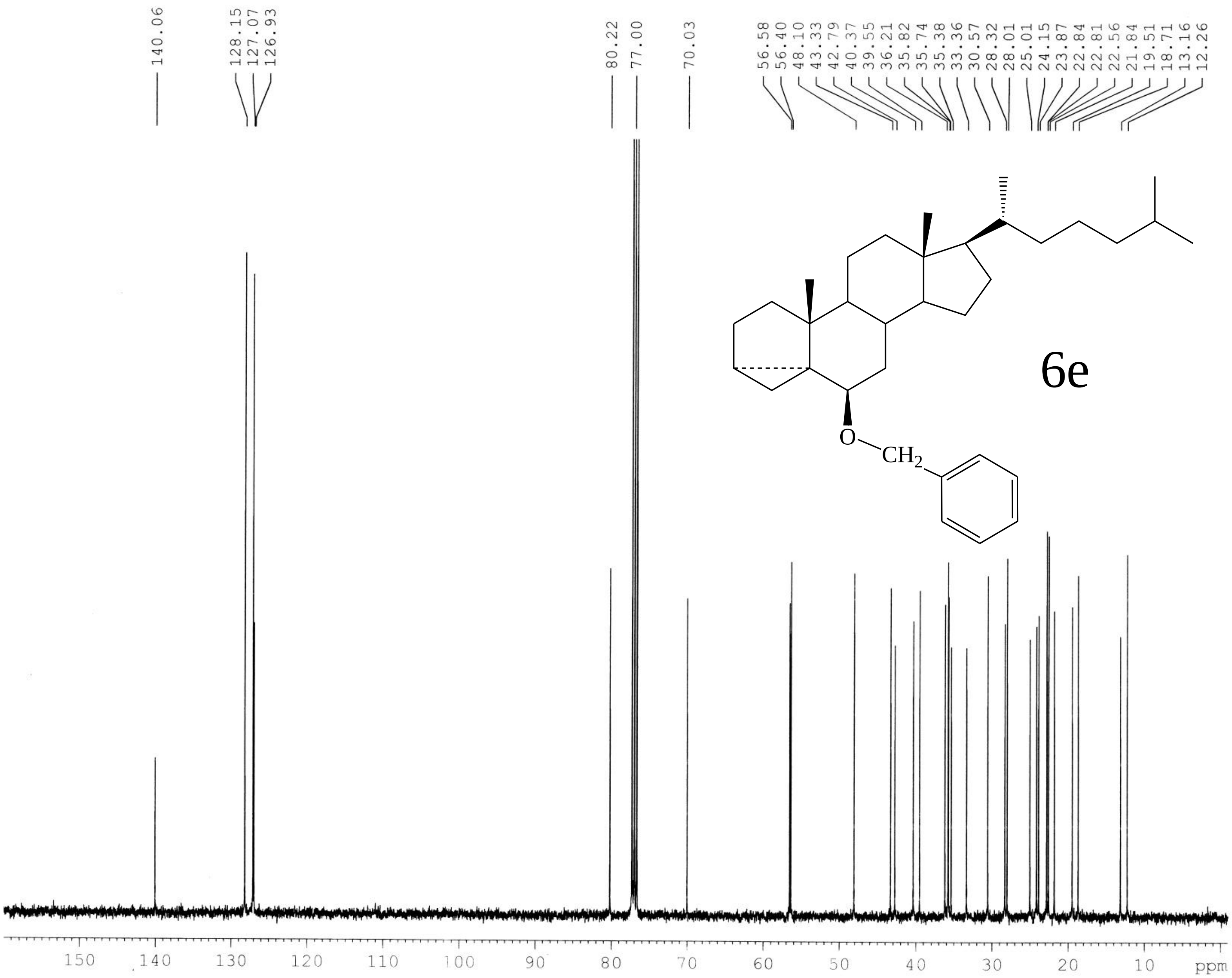

6e

Current Data Parameters
NAME EM Eve18
EXPNO 2
PROCNO 1

F2 - Acquisition Parameters
Date_ 20130314
Time 15.16
INSTRUM spect
PROBHD 5 mm PABBO BB-
PULPROG zgpg30
TD 65536
SOLVENT CDCl3
NS 384
DS 4
SWH 27173.912 Hz
FIDRES 0.414641 Hz
AQ 1.2059124 sec
RG 2050
DW 18.400 usec
DE 6.00 usec
TE 303.0 K
D1 2.00000000 sec
d11 0.03000000 sec
DELTA 1.89999998 sec
TD0 1

======== CHANNEL f1 ========
NUC1 13C
P1 12.25 usec
PL1 -1.00 dB
SFO1 100.6288660 MHz

======== CHANNEL f2 ========
CPDPRG2 waltz16
NUC2 1H
PCPD2 100.00 usec
PL2 -3.00 dB
PL12 13.65 dB
PL13 18.00 dB
SFO2 400.1516006 MHz

F2 - Processing parameters
SI 32768
SF 100.6177986 MHz
WDW EM
SSB 0
LB 1.00 Hz
GB 0
PC 1.40

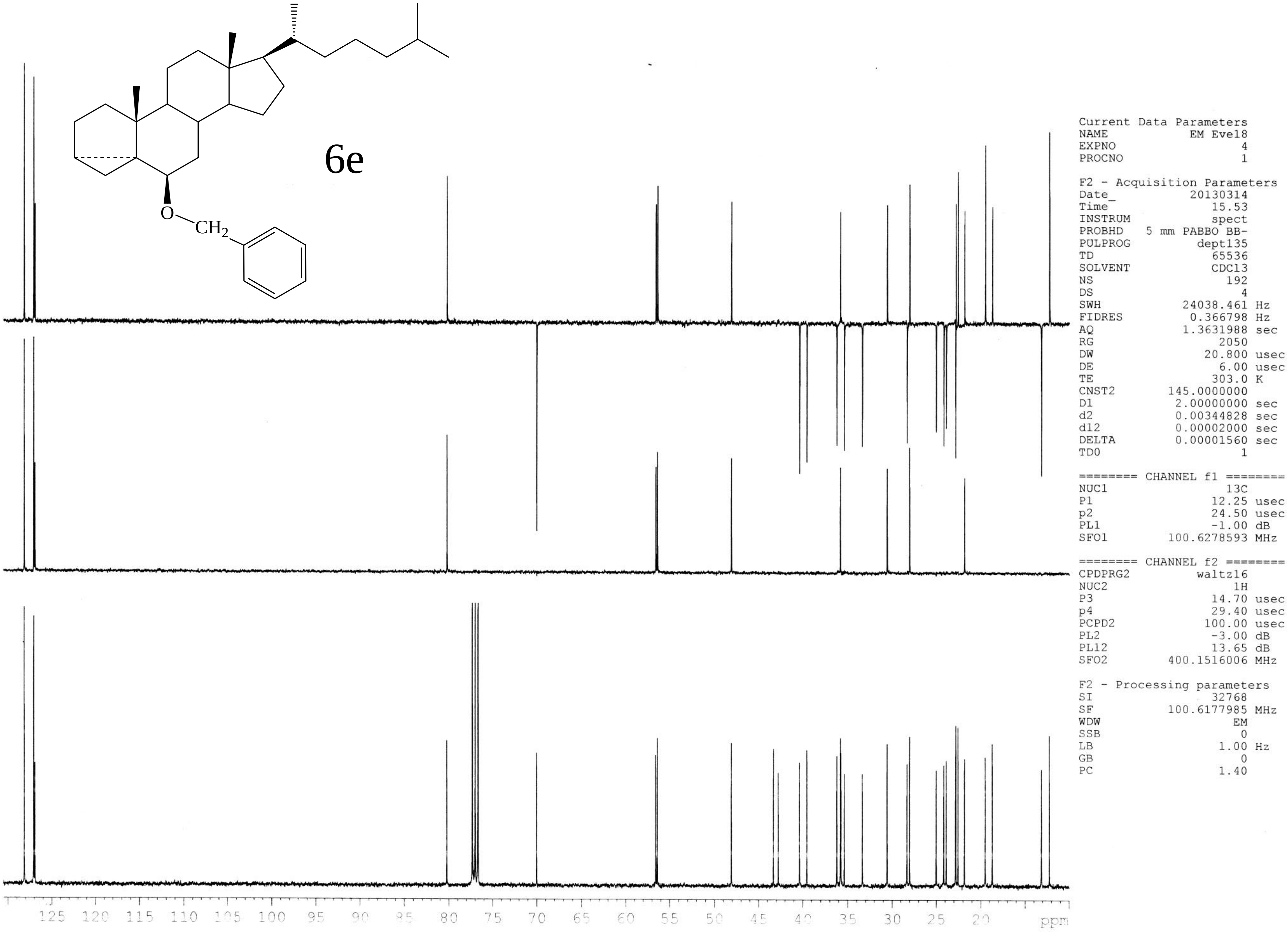
6e
O
CH2
Current Data Parameters
NAME EM Eve18
EXPNO 4
PROCNO 1
F2 - Acquisition Parameters
Date_ 20130314
Time 15.53
INSTRUM spect
PROBHD 5 mm PABBO BB-
PULPROG dept135
TD 65536
SOLVENT CDCl3
NS 192
DS 4
SWH 24038.461 Hz
FIDRES 0.366798 Hz
AQ 1.3631988 sec
RG 2050
DW 20.800 usec
DE 6.00 usec
TE 303.0 K
CNST2 145.0000000
D1 2.00000000 sec
d2 0.00344828 sec
d12 0.00002000 sec
DELTA 0.00001560 sec
TD0 1
======== CHANNEL f1 ========
NUC1 13C
P1 12.25 usec
p2 24.50 usec
PL1 -1.00 dB
SFO1 100.6278593 MHz
======== CHANNEL f2 ========
CPDPRG2 waltz16
NUC2 1H
P3 14.70 usec
p4 29.40 usec
PCPD2 100.00 usec
PL2 -3.00 dB
PL12 13.65 dB
SFO2 400.1516006 MHz
F2 - Processing parameters
SI 32768
SF 100.6177985 MHz
WDW EM
SSB 0
LB 1.00 Hz
GB 0
PC 1.40
125 120 115 110 105 100 95 90 85 80 75 70 65 60 55 50 45 40 35 30 25 20 ppm

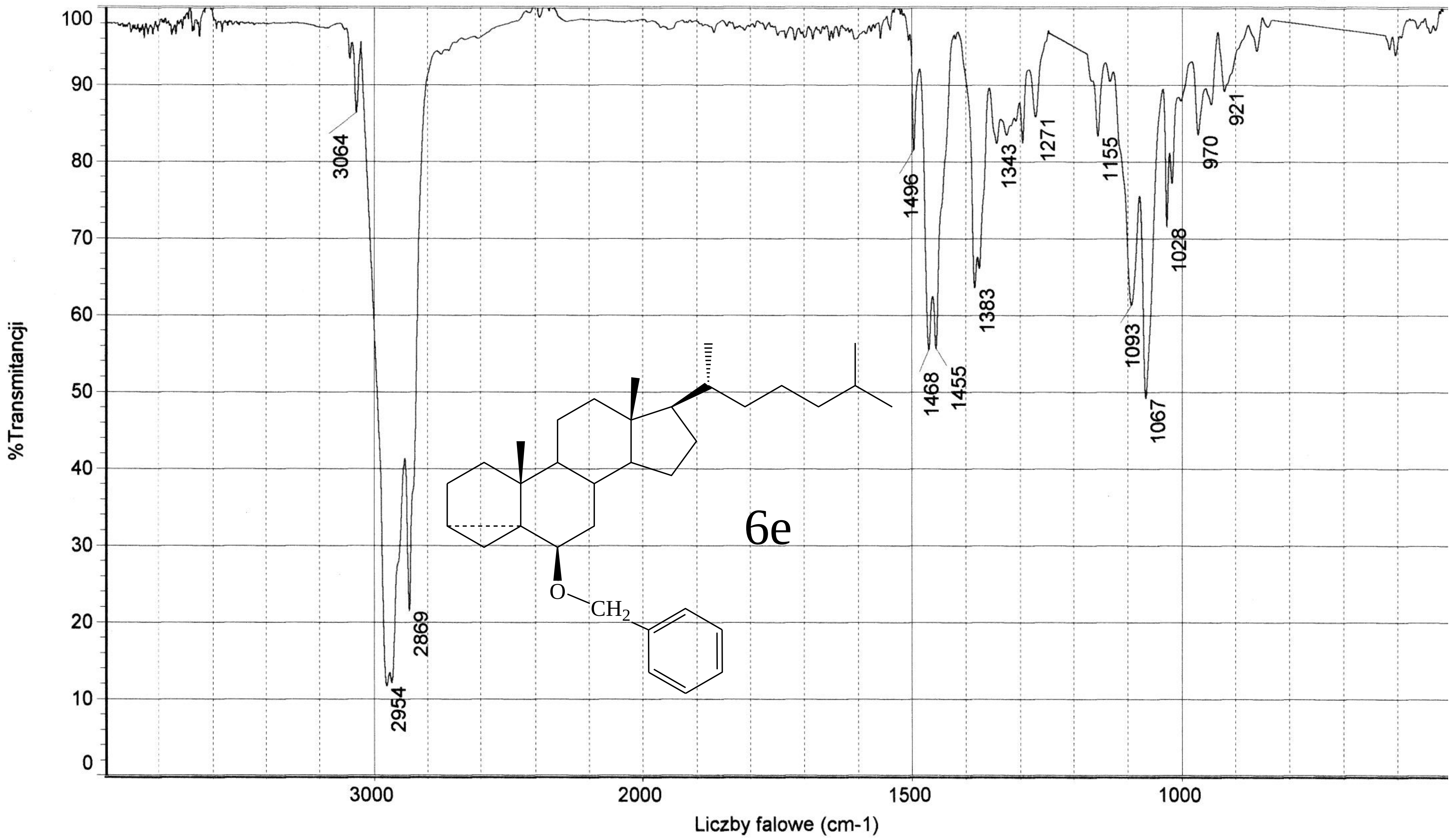
%Transmitancji
Liczby falowe (cm-1)
100
90
80
70
60
50
40
30
20
10
0
3000
2000
1500
1000
3064
2954
2869
1496
1468
1455
1383
1343
1271
1155
1093
1067
1028
970
921
O
CH2
6e

A. Tomkiel **AUTOSPEC** **19-Mar-2013 08:51:05**

Eve19

ub_at0865 35 (2.775) Cm (31:38) Magnet EI+ 3.90e4

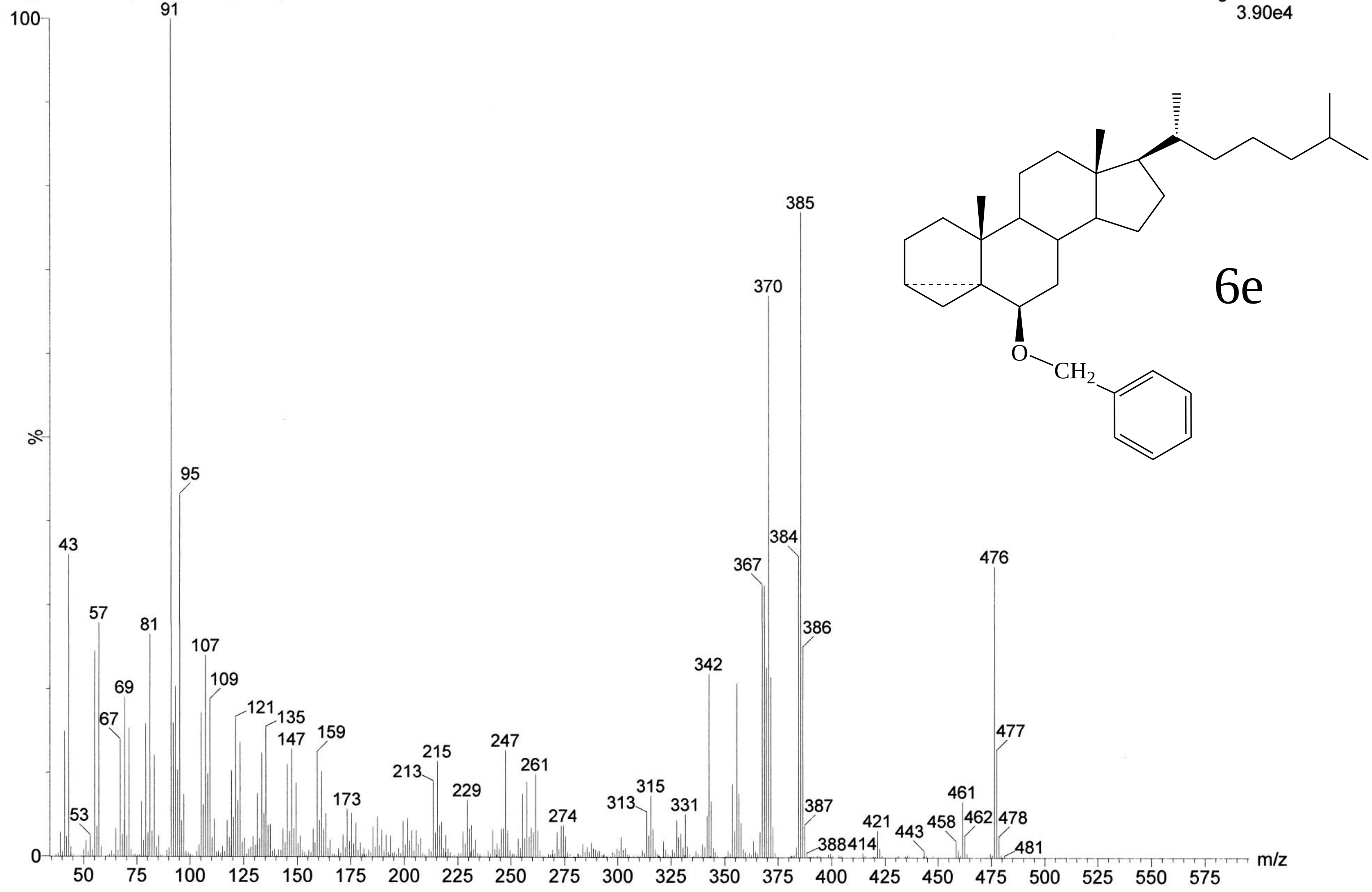

6e

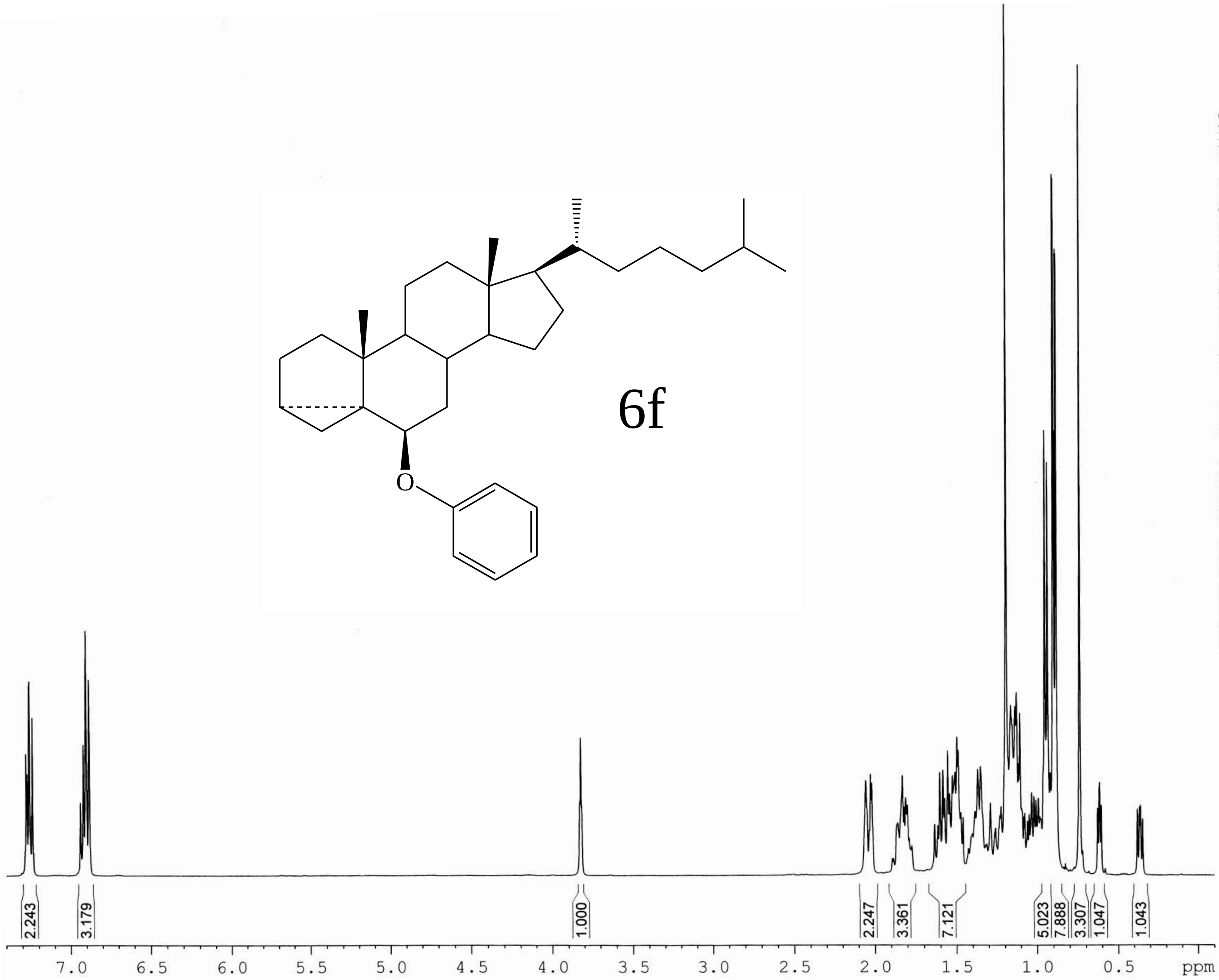


```
Current Data Parameters
NAME              ABd XI-1
EXPNO                    1
PROCNO                   1

F2 - Acquisition Parameters
Date_             20110120
Time                 15.00
INSTRUM              spect
PROBHD   5 mm PABBO BB-
PULPROG               zg30
TD                   65536
SOLVENT              CDCl3
NS                      32
DS                       0
SWH               8223.685 Hz
FIDRES            0.125483 Hz
AQ               3.9846387 sec
RG                      57
DW                  60.800 usec
DE                    8.00 usec
TE                   297.6 K
D1              1.00000000 sec
TD0                      1

======== CHANNEL f1 ========
NUC1                    1H
P1                   11.15 usec
PL1                  -3.00 dB
SFO1           400.1524711 MHz

F2 - Processing parameters
SI                   32768
SF             400.1500000 MHz
WDW                     GM
SSB                      0
LB                   -0.20 Hz
GB                     0.2
PC                    1.00
```

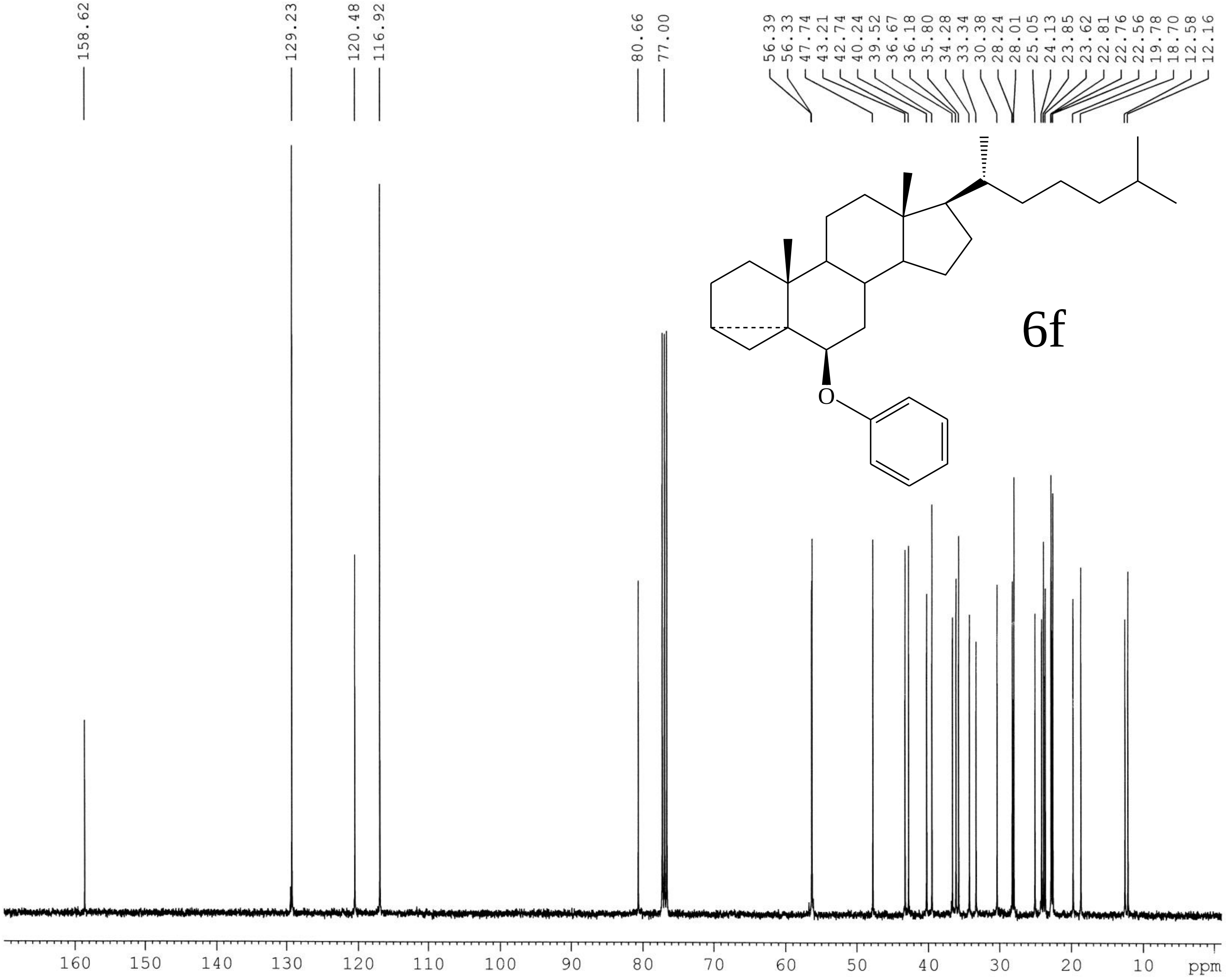


```
Current Data Parameters
NAME            ABd XI-1
EXPNO                  2
PROCNO                 1

F2 - Acquisition Parameters
Date_           20110329
Time               12.56
INSTRUM            spect
PROBHD   5 mm PABBO BB-
PULPROG          zgpg30
TD                 65536
SOLVENT            CDCl3
NS                   576
DS                     4
SWH            27173.912 Hz
FIDRES          0.414641 Hz
AQ             1.2059124 sec
RG                  1030
DW                18.400 usec
DE                  6.00 usec
TE                 299.1 K
D1            2.00000000 sec
d11           0.03000000 sec
DELTA         1.89999998 sec
TD0                    1

======== CHANNEL f1 ========
NUC1                 13C
P1                 11.00 usec
PL1                -1.00 dB
SFO1         100.6288660 MHz

======== CHANNEL f2 ========
CPDPRG2          waltz16
NUC2                  1H
PCPD2             100.00 usec
PL2                -3.00 dB
PL12               13.65 dB
PL13               18.00 dB
SFO2         400.1516006 MHz

F2 - Processing parameters
SI                 32768
SF           100.6178008 MHz
WDW                   EM
SSB                    0
LB                  1.00 Hz
GB                     0
PC                  0.10
```

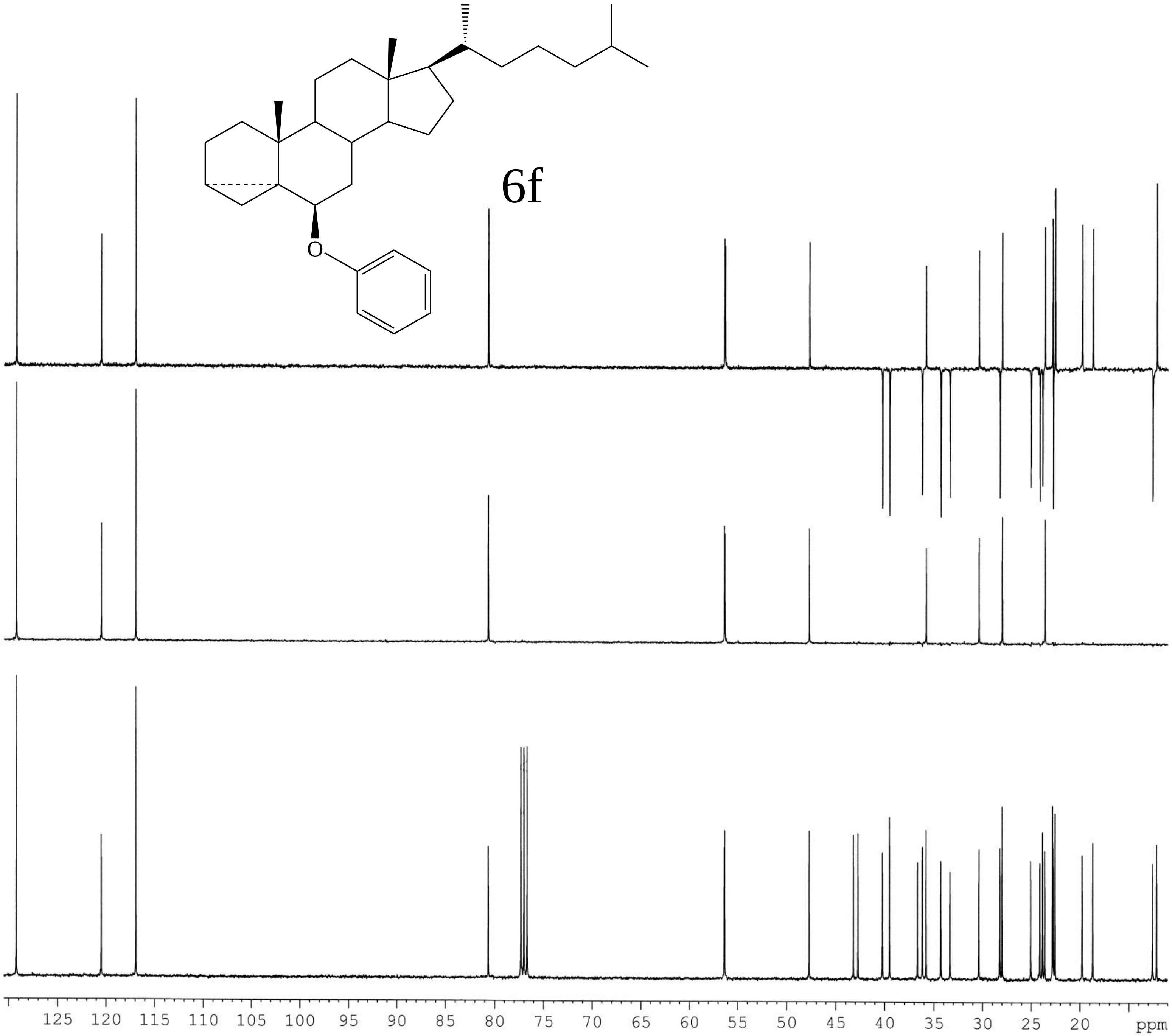


```
Current Data Parameters
NAME            ABd XI-1
EXPNO                  4
PROCNO                 1

F2 - Acquisition Parameters
Date_           20110329
Time               13.41
INSTRUM            spect
PROBHD   5 mm PABBO BB-
PULPROG          dept135
TD                 65536
SOLVENT            CDCl3
NS                   192
DS                     4
SWH            24038.461 Hz
FIDRES          0.366798 Hz
AQ             1.3631988 sec
RG                  2050
DW                20.800 usec
DE                  6.00 usec
TE                 299.7 K
CNST2        145.0000000
D1            2.00000000 sec
d2            0.00344828 sec
d12           0.00002000 sec
DELTA         0.00001401 sec
TD0                    1

======== CHANNEL f1 ========
NUC1                 13C
P1                 11.00 usec
p2                 22.00 usec
PL1                -1.00 dB
SFO1         100.6278593 MHz

======== CHANNEL f2 ========
CPDPRG2          waltz16
NUC2                  1H
P3                 14.70 usec
p4                 29.40 usec
PCPD2             100.00 usec
PL2                -3.00 dB
PL12               13.65 dB
SFO2         400.1516006 MHz

F2 - Processing parameters
SI                 32768
SF           100.6178008 MHz
WDW                   EM
SSB                    0
LB                  1.00 Hz
GB                     0
PC                  0.10
```

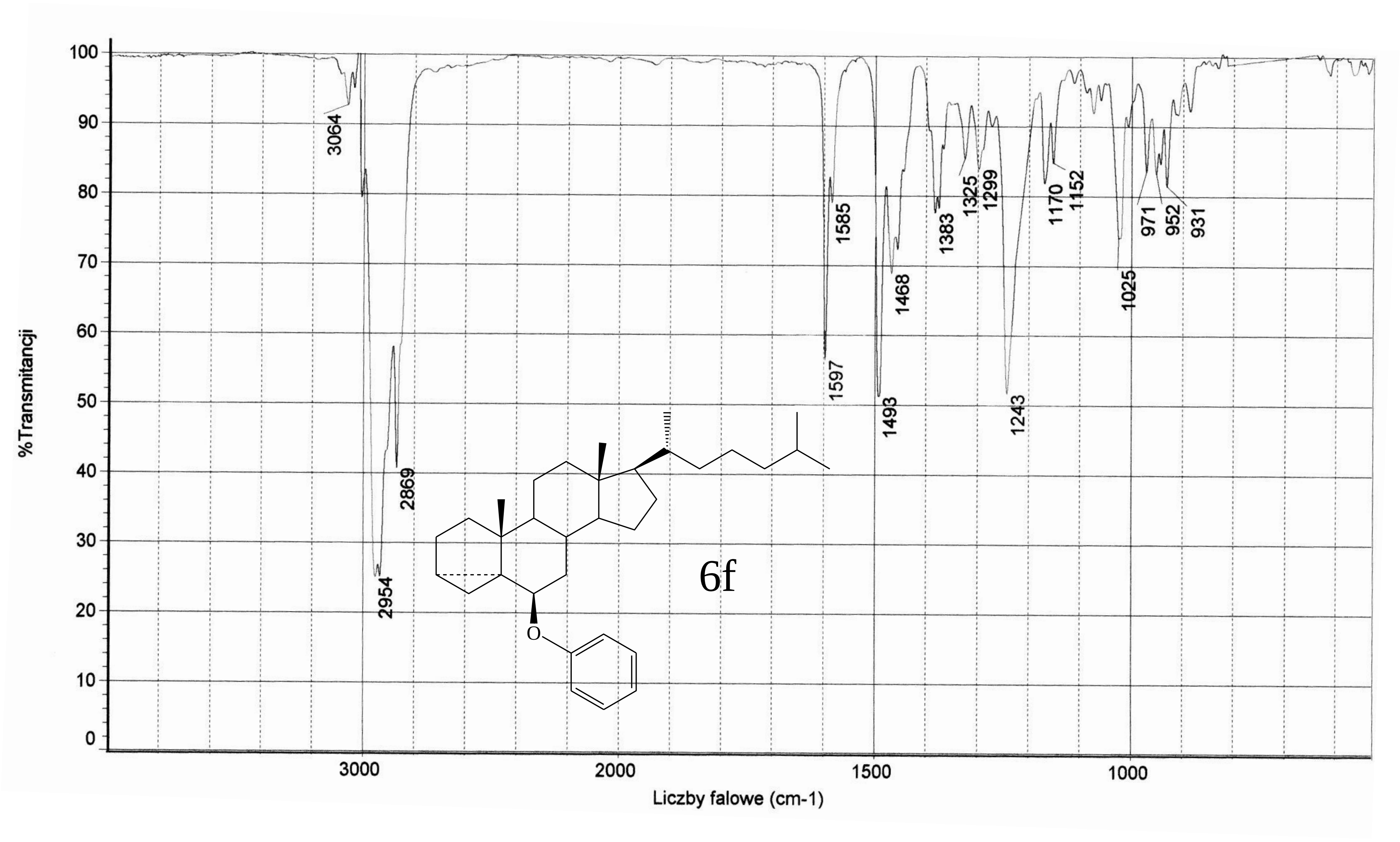

%Transmitancji
100
90
80
70
60
50
40
30
20
10
0
3000
2000
1500
1000
Liczby falowe (cm-1)
3064
2954
2869
1597
1585
1493
1468
1383
1325
1299
1243
1170
1152
1025
971
952
931
O
6f

AUTOSPEC

14-Apr-2014 10:46:12

eter fenylowy w6

ub_jm1101 70 (5.698) Cm (70:77)

Magnet EI+
1.00e4

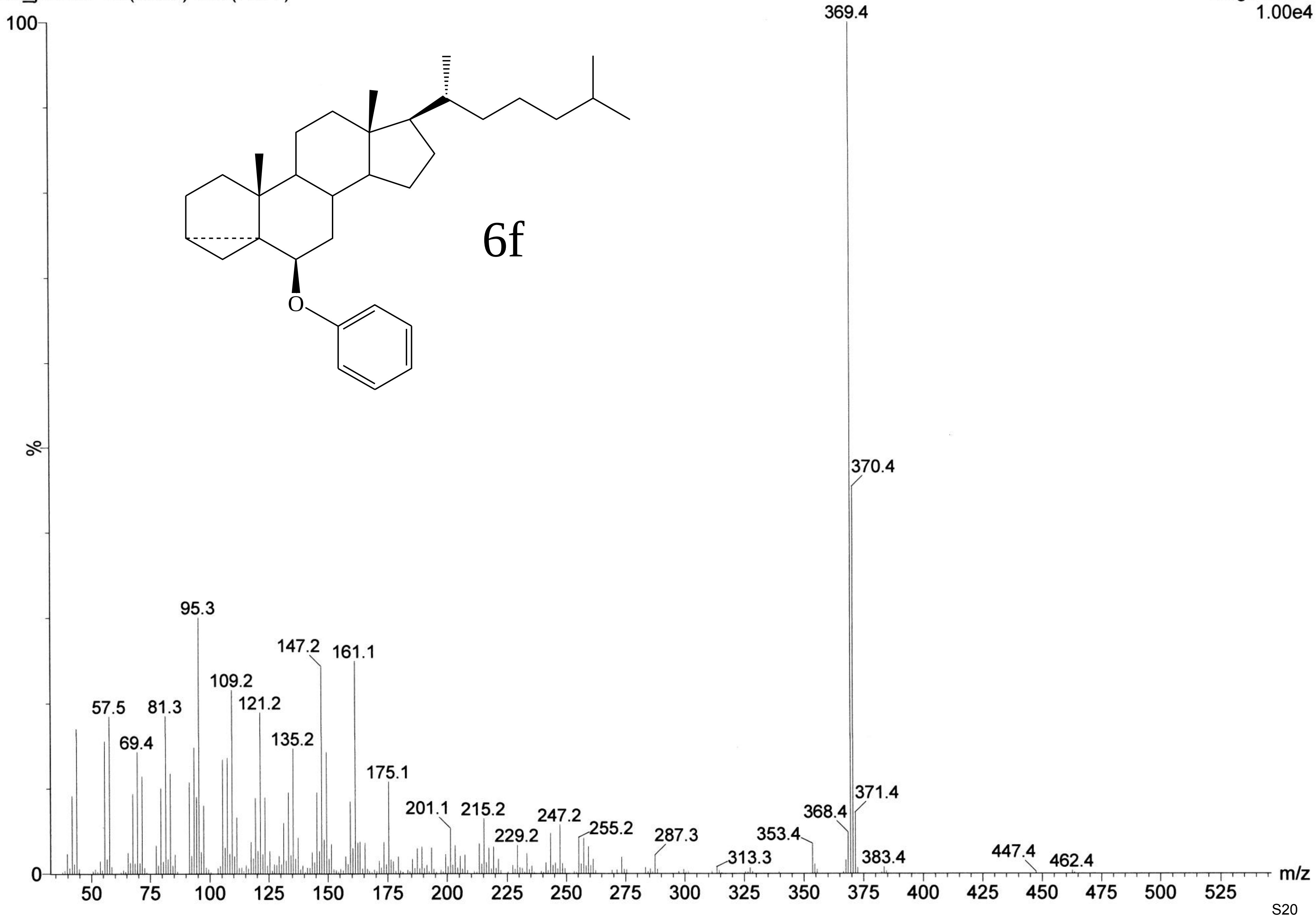

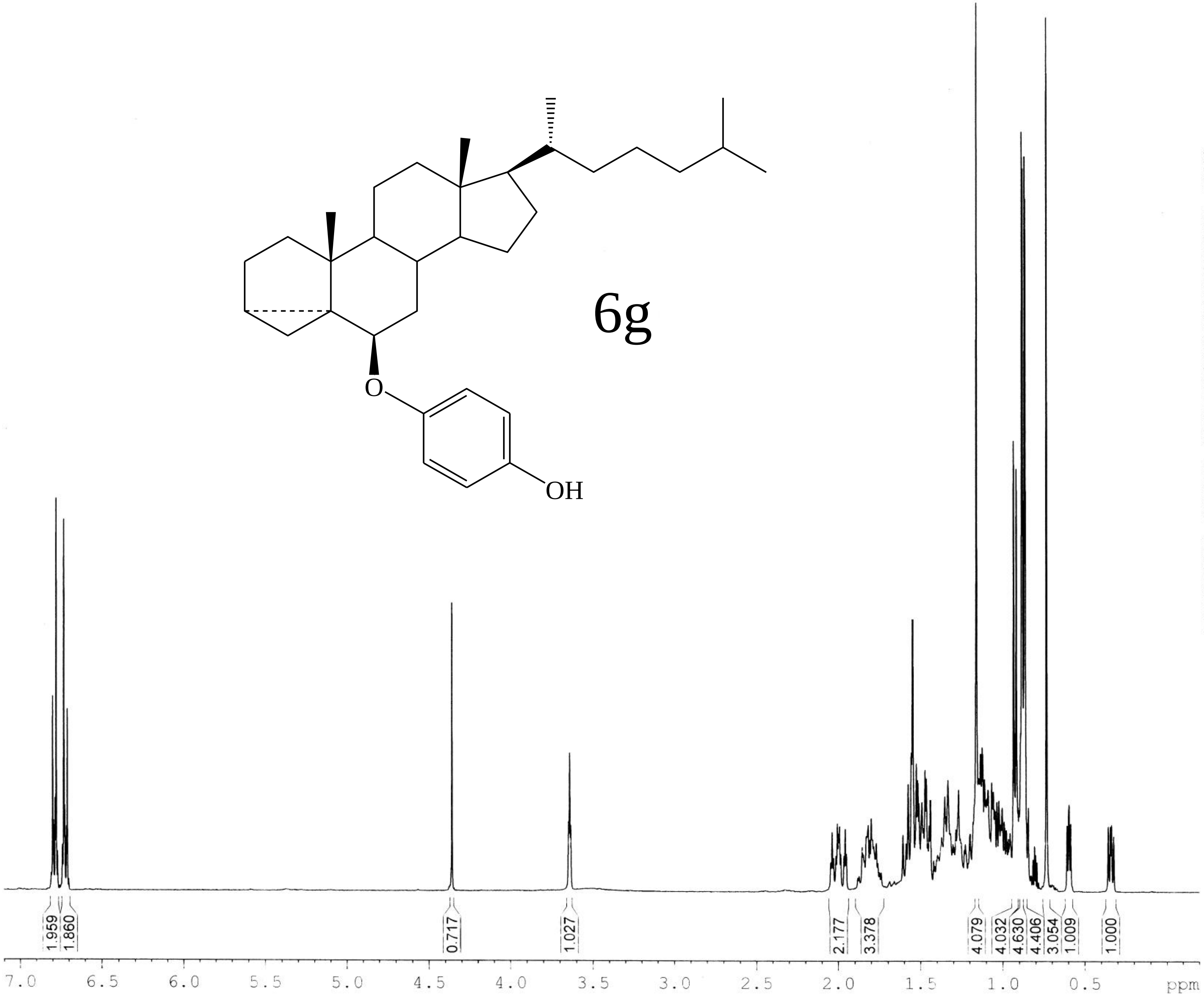


```
Current Data Parameters
NAME     EM 6-et.h.chinonowy cz.
EXPNO                  1
PROCNO                 1

F2 - Acquisition Parameters
Date_           20130411
Time               11.53
INSTRUM            spect
PROBHD   5 mm PABBO BB-
PULPROG             zg30
TD                 65536
SOLVENT            CDCl3
NS                   192
DS                     0
SWH             8223.685 Hz
FIDRES          0.125483 Hz
AQ             3.9846387 sec
RG                   287
DW                60.800 usec
DE                  8.00 usec
TE                 673.2 K
D1            1.00000000 sec
TD0                    1

======== CHANNEL f1 ========
NUC1                  1H
P1                 30.00 usec
PL1                -3.00 dB
SFO1         400.1524711 MHz

F2 - Processing parameters
SI                 32768
SF           400.1500000 MHz
WDW                   GM
SSB                    0
LB                 -0.20 Hz
GB                   0.2
PC                  1.00
```

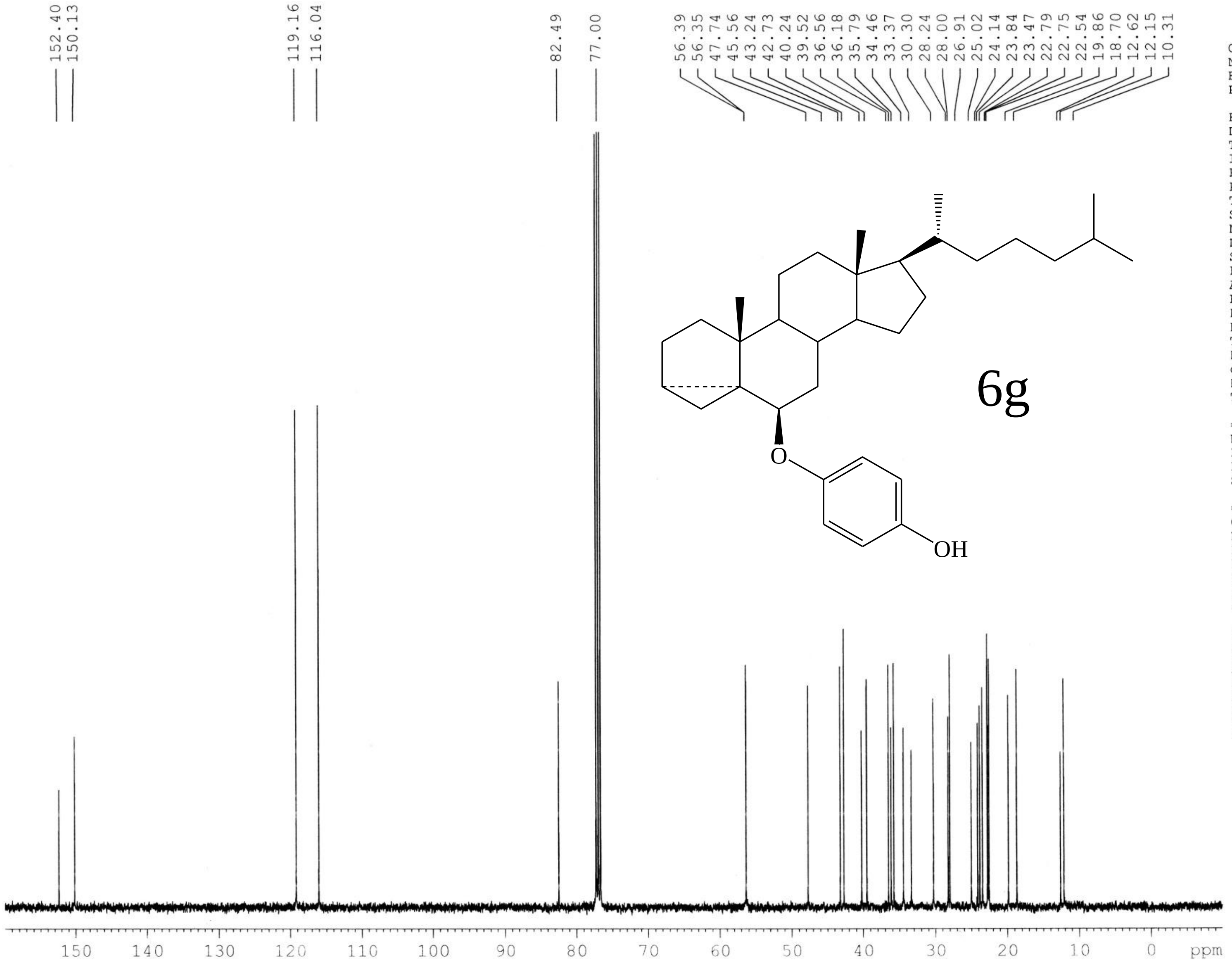


```
Current Data Parameters
NAME      EM Eve19czysty
EXPNO                  2
PROCNO                 1

F2 - Acquisition Parameters
Date_           20130315
Time               13.38
INSTRUM            spect
PROBHD   5 mm PABBO BB-
PULPROG          zgpg30
TD                 65536
SOLVENT            CDCl3
NS                   800
DS                     4
SWH           27173.912 Hz
FIDRES         0.414641 Hz
AQ            1.2059124 sec
RG                  2050
DW                18.400 usec
DE                  6.00 usec
TE                 303.2 K
D1            2.00000000 sec
d11           0.03000000 sec
DELTA         1.89999998 sec
TD0                    1

======== CHANNEL f1 ========
NUC1                 13C
P1                 12.25 usec
PL1                -1.00 dB
SFO1         100.6288660 MHz

======== CHANNEL f2 ========
CPDPRG2          waltz16
NUC2                  1H
PCPD2             100.00 usec
PL2                -3.00 dB
PL12               13.65 dB
PL13               18.00 dB
SFO2         400.1516006 MHz

F2 - Processing parameters
SI                 32768
SF           100.6177986 MHz
WDW                   EM
SSB                    0
LB                  1.00 Hz
GB                     0
PC                  0.10
```

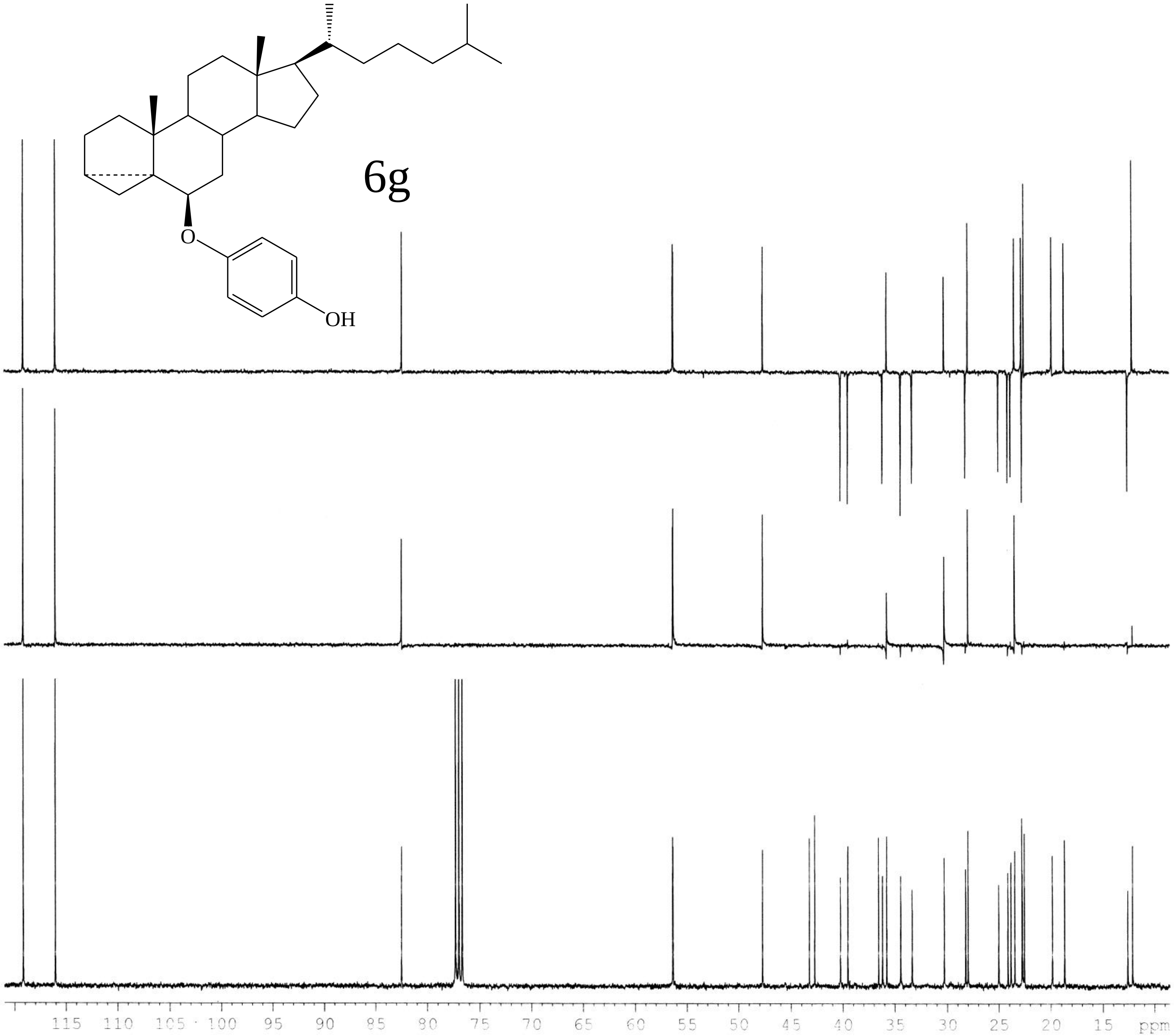


```
Current Data Parameters
NAME     EM Eve19czysty
EXPNO              4
PROCNO             1

F2 - Acquisition Parameters
Date_          20130315
Time              15.06
INSTRUM           spect
PROBHD   5 mm PABBO BB-
PULPROG         dept135
TD                65536
SOLVENT           CDCl3
NS                  320
DS                    4
SWH           24038.461 Hz
FIDRES         0.366798 Hz
AQ            1.3631988 sec
RG                 2050
DW               20.800 usec
DE                 6.00 usec
TE                303.0 K
CNST2       145.0000000
D1           2.00000000 sec
d2           0.00344828 sec
d12          0.00002000 sec
DELTA        0.00001560 sec
TD0                   1

======== CHANNEL f1 ========
NUC1                13C
P1                12.25 usec
p2                24.50 usec
PL1               -1.00 dB
SFO1        100.6278593 MHz

======== CHANNEL f2 ========
CPDPRG2         waltz16
NUC2                 1H
P3                14.70 usec
p4                29.40 usec
PCPD2            100.00 usec
PL2               -3.00 dB
PL12              13.65 dB
SFO2        400.1516006 MHz

F2 - Processing parameters
SI                32768
SF          100.6177987 MHz
WDW                  EM
SSB                   0
LB                 1.00 Hz
GB                    0
PC                 0.10
```

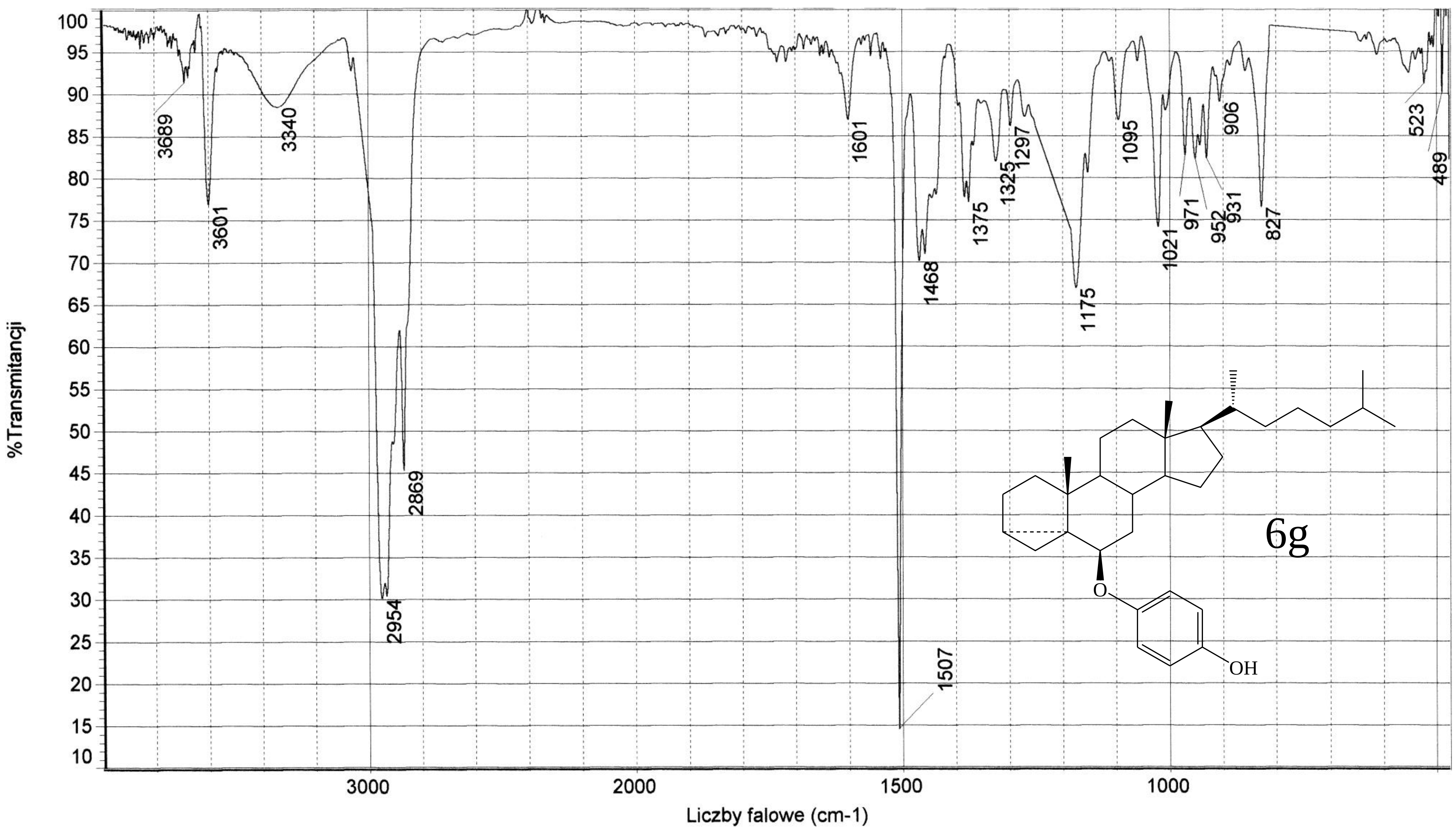
%Transmitancji
Liczby falowe (cm-1)
3689
3601
3340
2954
2869
1601
1507
1468
1375
1325
1297
1175
1095
1021
971
952
931
906
827
523
489
O
OH
6g

AUTOSPEC

14-Apr-2014 11:03:43

eter chinonowy w6

ub_jm1102 19 (1.546) Cm (19:27-3:6)

Magnet EI+
4.43e3

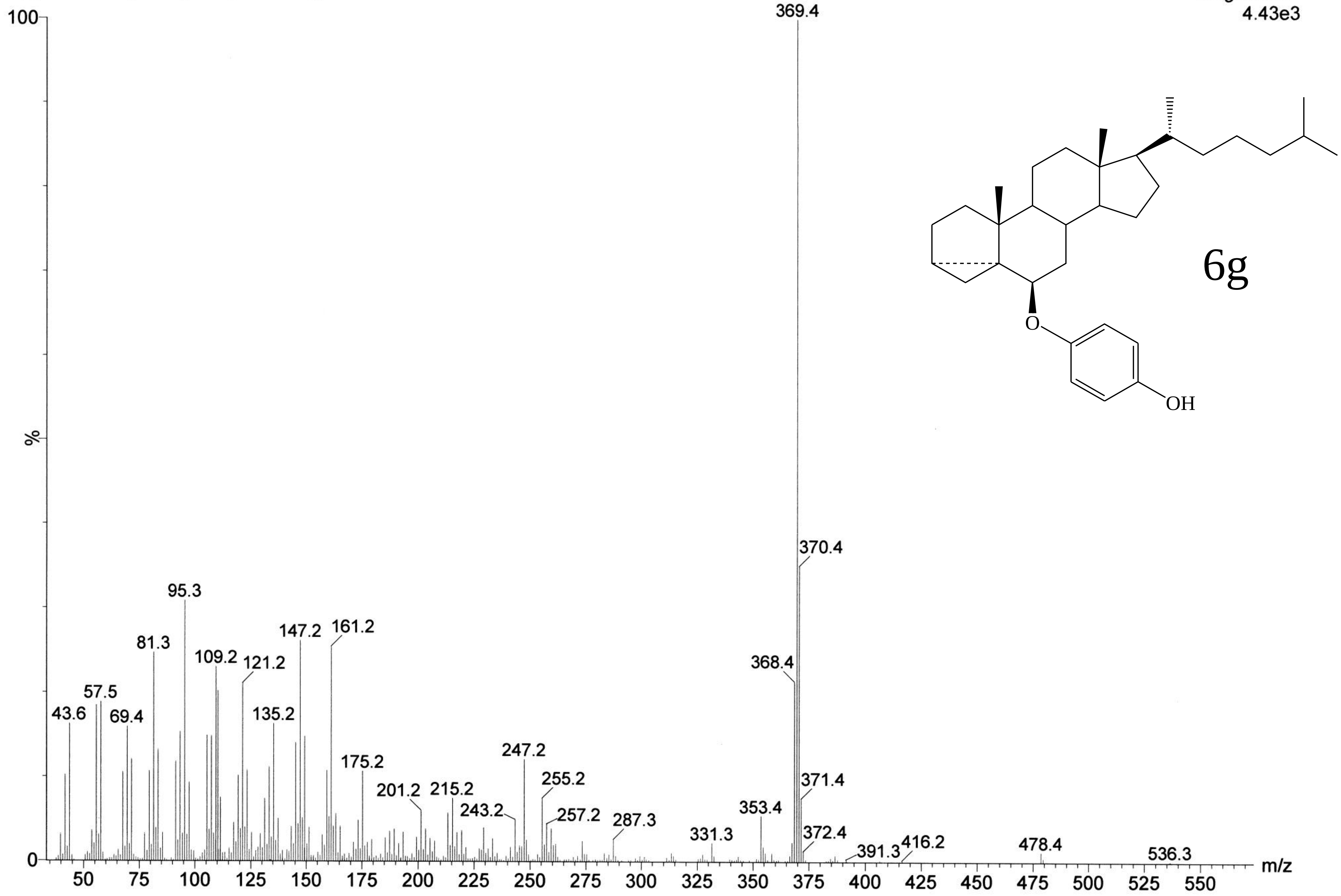

6g

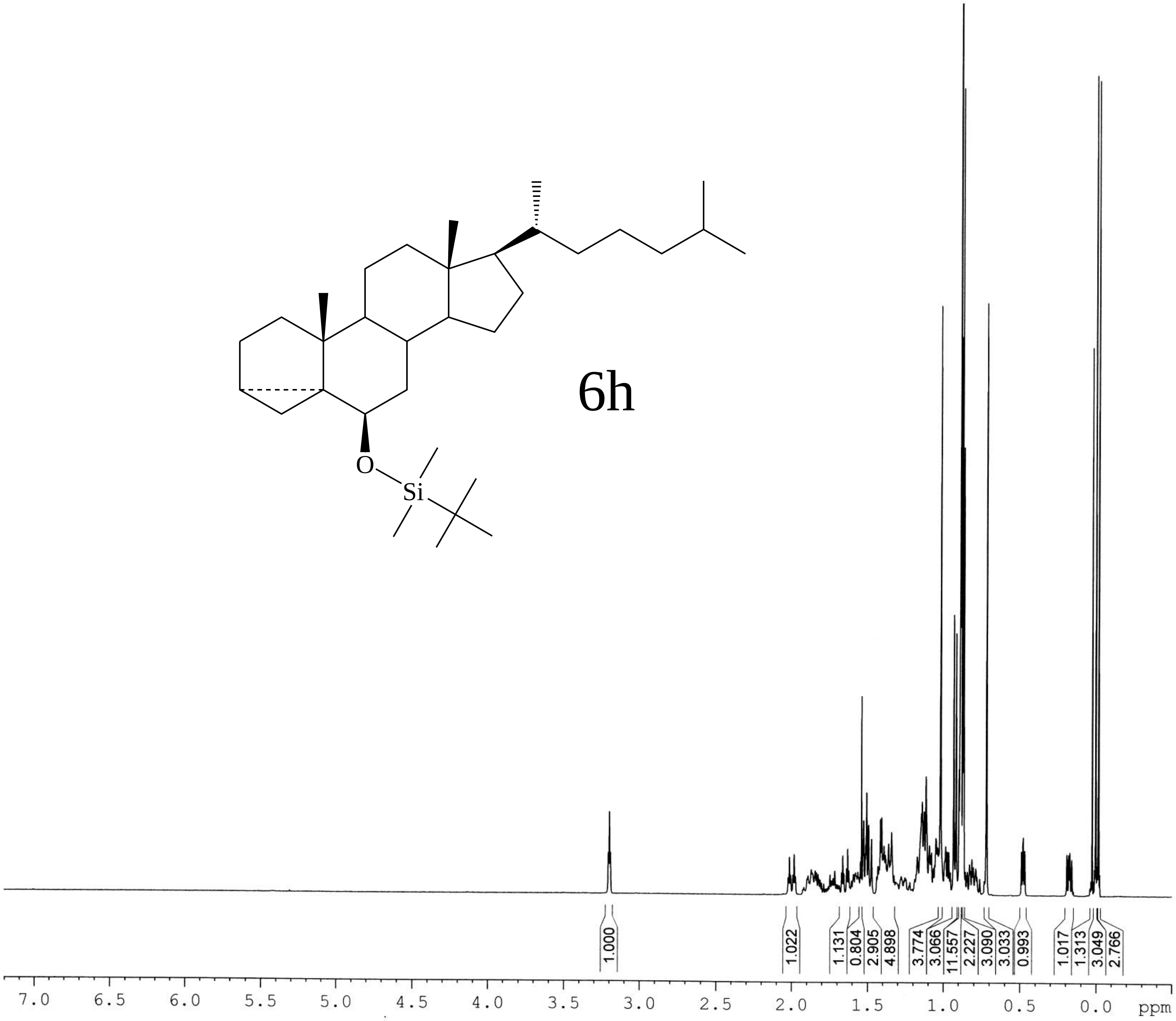

Current Data Parameters
NAME ABd VIII
EXPNO 1
PROCNO 1

F2 - Acquisition Parameters
Date_ 20101220
Time 11.19
INSTRUM spect
PROBHD 5 mm PABBO BB-
PULPROG zg30
TD 65536
SOLVENT CDCl3
NS 128
DS 0
SWH 8223.685 Hz
FIDRES 0.125483 Hz
AQ 3.9846387 sec
RG 228
DW 60.800 usec
DE 8.00 usec
TE 299.4 K
D1 1.00000000 sec
TD0 1

======== CHANNEL f1 ========
NUC1 1H
P1 11.15 usec
PL1 -3.00 dB
SFO1 400.1524711 MHz

F2 - Processing parameters
SI 32768
SF 400.1500000 MHz
WDW GM
SSB 0
LB -0.20 Hz
GB 0.2
PC 1.00

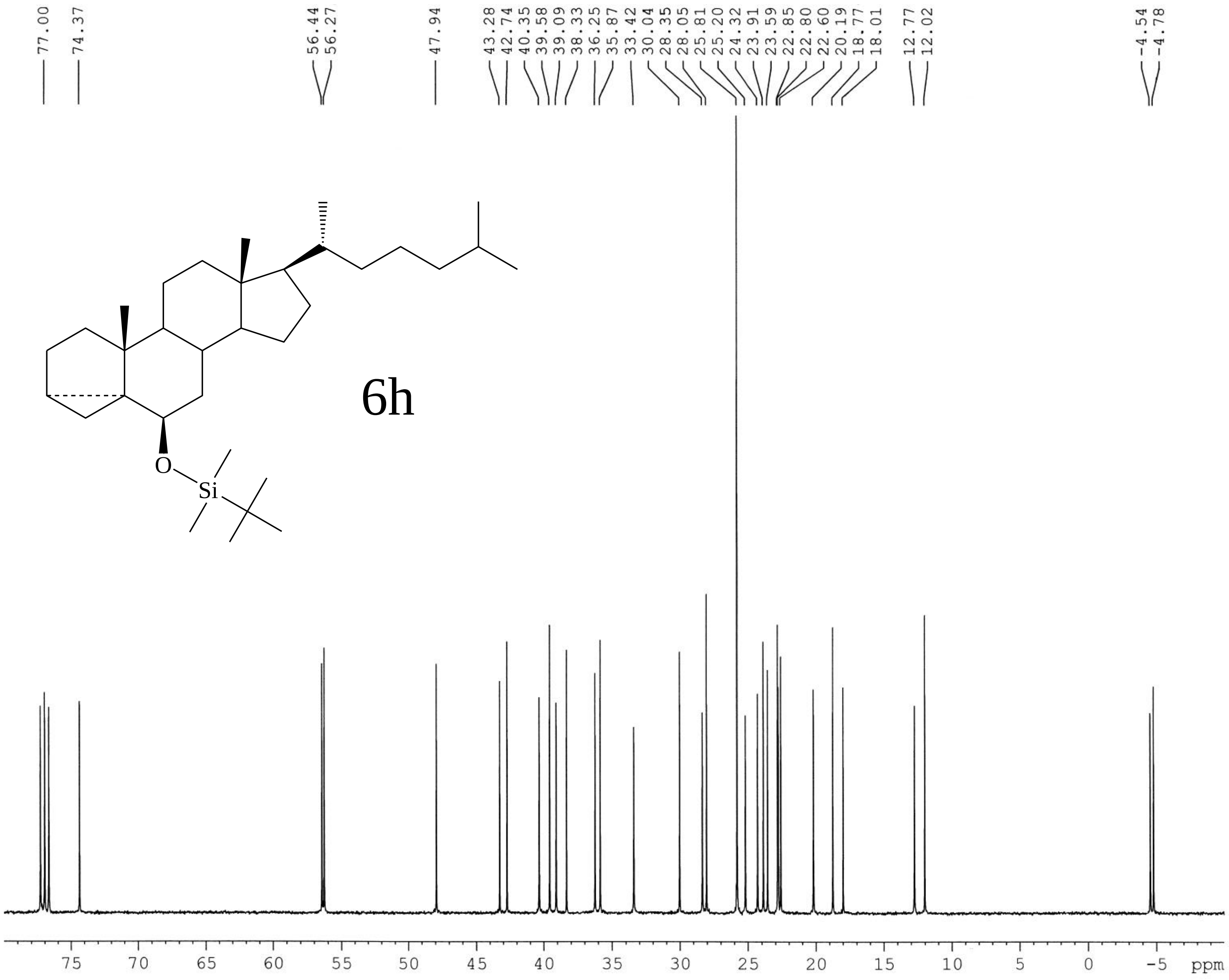


```
Current Data Parameters
NAME              ABd VIII
EXPNO                    2
PROCNO                   1

F2 - Acquisition Parameters
Date_             20110309
Time                 16.48
INSTRUM              spect
PROBHD   5 mm PABBO BB-
PULPROG            zgpg30
TD                   65536
SOLVENT              CDCl3
NS                     448
DS                       4
SWH              27173.912 Hz
FIDRES            0.414641 Hz
AQ               1.2059124 sec
RG                    1030
DW                  18.400 usec
DE                    6.00 usec
TE                   299.9 K
D1              2.00000000 sec
d11             0.03000000 sec
DELTA           1.89999998 sec
TD0                      1

======== CHANNEL f1 ========
NUC1                   13C
P1                   11.00 usec
PL1                  -1.00 dB
SFO1           100.6288660 MHz

======== CHANNEL f2 ========
CPDPRG2            waltz16
NUC2                    1H
PCPD2               100.00 usec
PL2                  -3.00 dB
PL12                 13.65 dB
PL13                 18.00 dB
SFO2           400.1516006 MHz

F2 - Processing parameters
SI                   32768
SF             100.6177950 MHz
WDW                     EM
SSB                      0
LB                    1.00 Hz
GB                       0
PC                    1.40
```

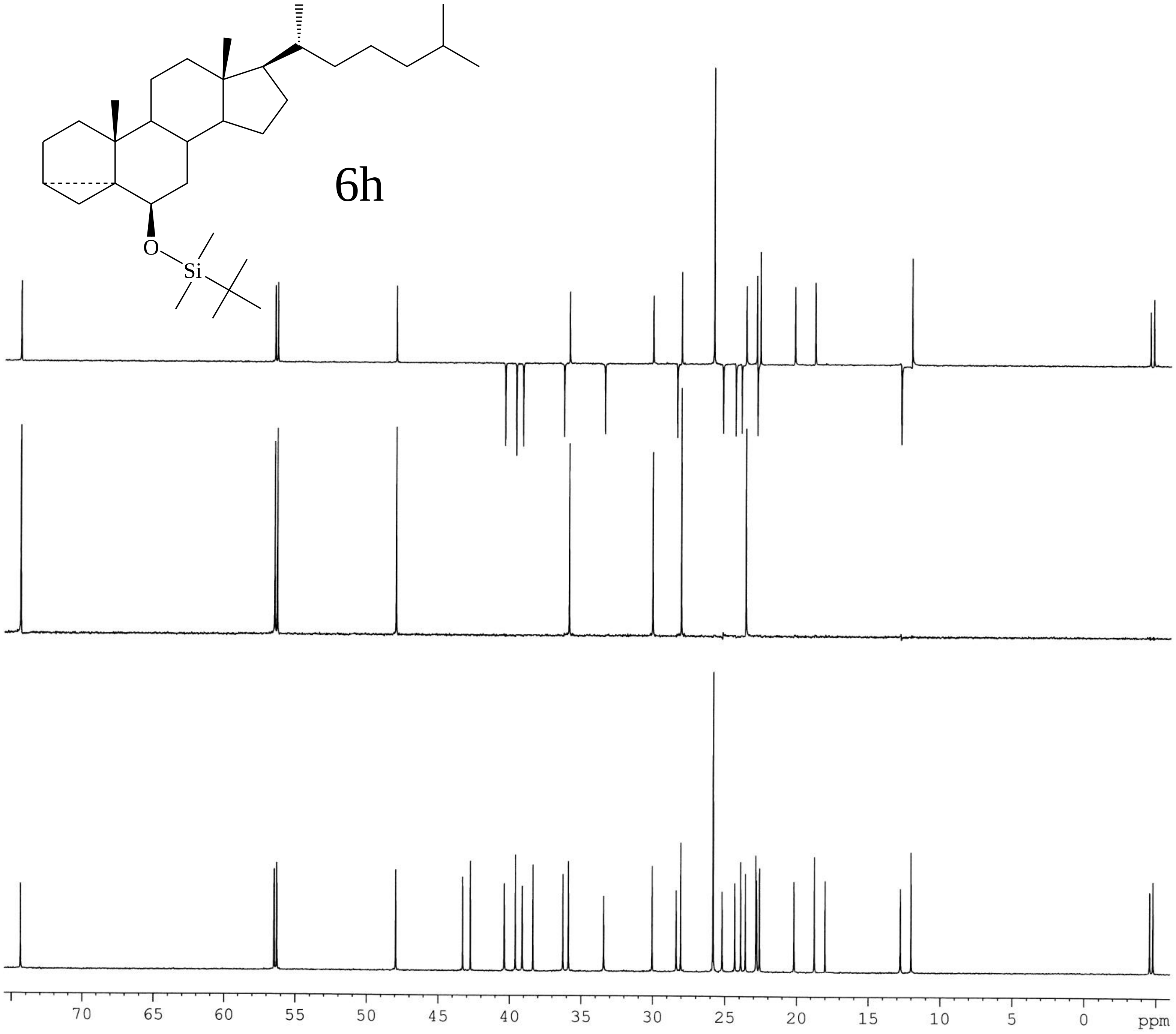


```
Current Data Parameters
NAME            ABd VIII
EXPNO                  4
PROCNO                 1

F2 - Acquisition Parameters
Date_           20110309
Time               17.25
INSTRUM            spect
PROBHD   5 mm PABBO BB-
PULPROG          dept135
TD                 65536
SOLVENT            CDCl3
NS                   128
DS                     4
SWH            24038.461 Hz
FIDRES          0.366798 Hz
AQ             1.3631988 sec
RG                  1030
DW                20.800 usec
DE                  6.00 usec
TE                 299.8 K
CNST2        145.0000000
D1            2.00000000 sec
d2            0.00344828 sec
d12           0.00002000 sec
DELTA         0.00001401 sec
TD0                    1

======== CHANNEL f1 ========
NUC1                 13C
P1                 11.00 usec
p2                 22.00 usec
PL1                -1.00 dB
SFO1         100.6278593 MHz

======== CHANNEL f2 ========
CPDPRG2           waltz16
NUC2                  1H
P3                 14.70 usec
p4                 29.40 usec
PCPD2             100.00 usec
PL2                -3.00 dB
PL12               13.65 dB
SFO2         400.1516006 MHz

F2 - Processing parameters
SI                 32768
SF           100.6177949 MHz
WDW                   EM
SSB                    0
LB                  1.00 Hz
GB                     0
PC                  0.50
```

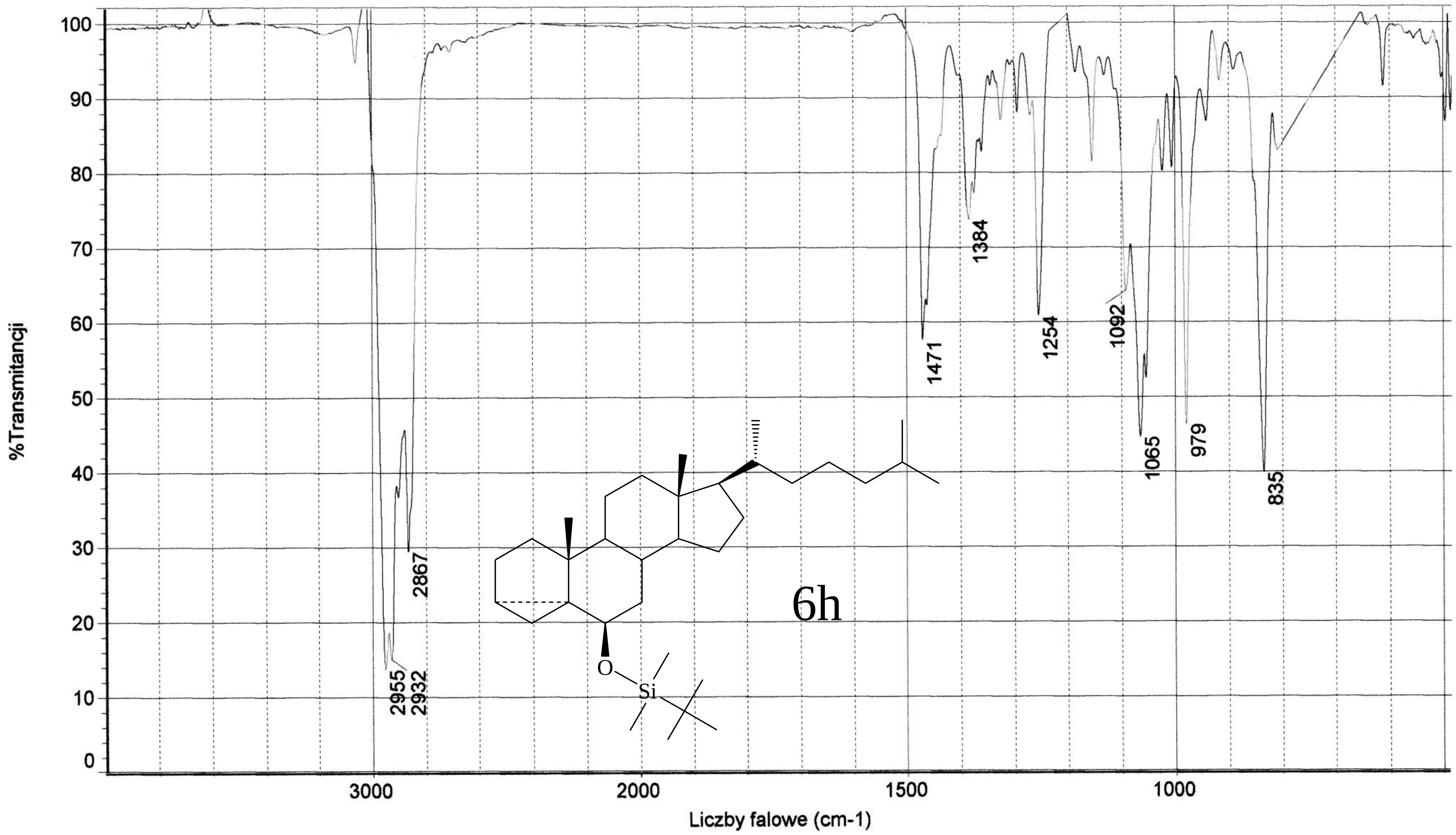

100
90
80
70
60
50
40
30
20
10
0
%Transmitancji
3000
2000
1500
1000
Liczby falowe (cm-1)
2955
2932
2867
1471
1384
1254
1092
1065
979
835
O
Si

6h

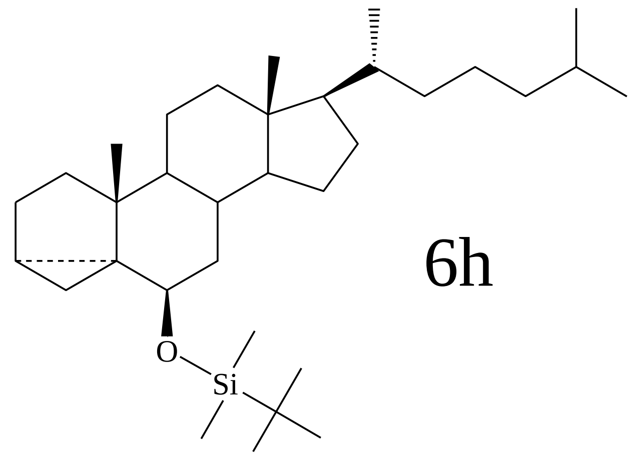

6h

A. Bd-VIII +HCOONa
LCT
9742_JWM_HCOONa 74 (1.233) Cm (1:94)

18-Jan-2011

TOF MS ES+
207

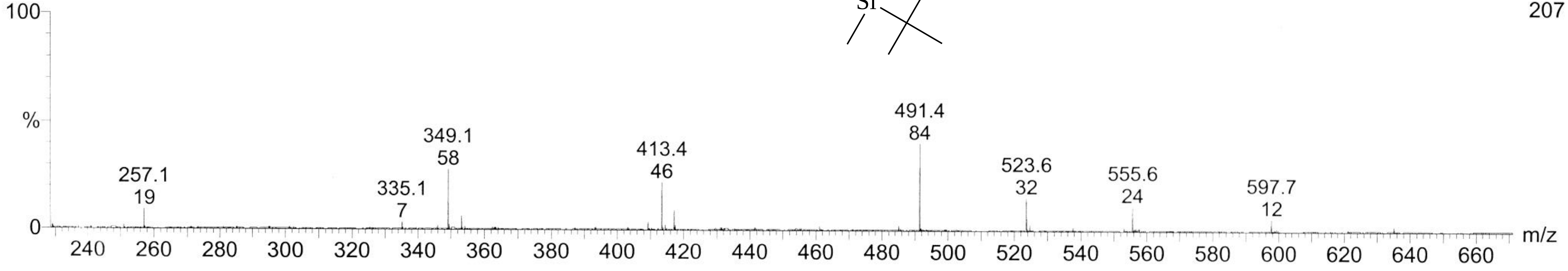

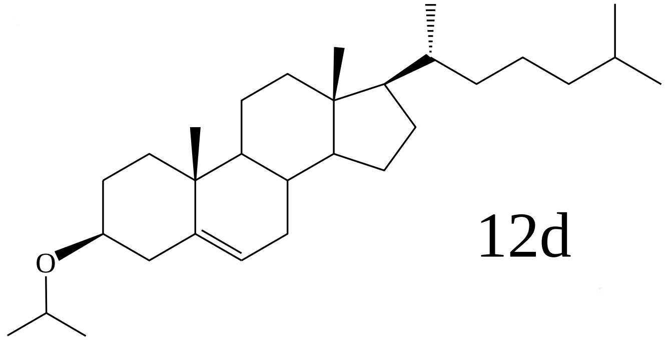

12d

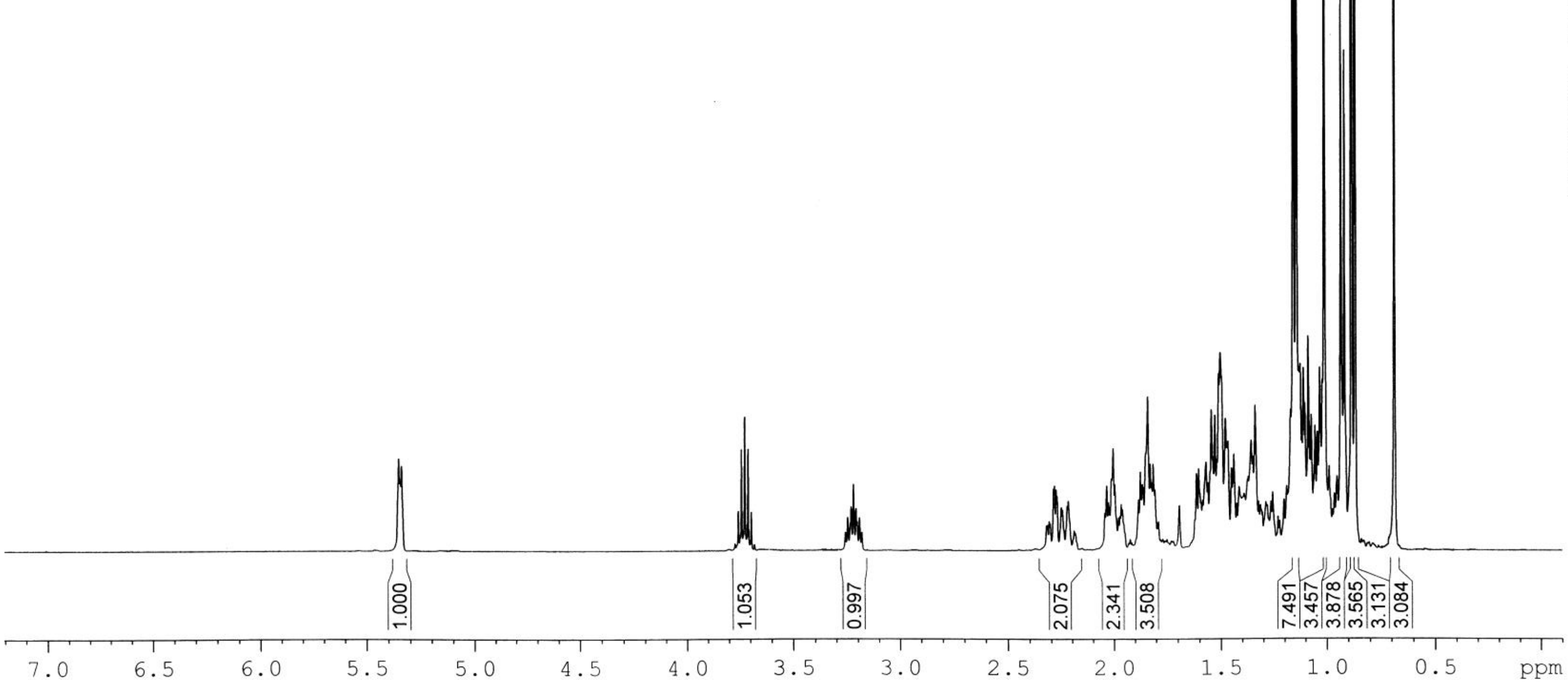


```
Current Data Parameters
NAME      AT eterizopropylowyw3
EXPNO                   1
PROCNO                  1

F2 - Acquisition Parameters
Date_            20140425
Time                16.10
INSTRUM             spect
PROBHD   5 mm PABBO BB-
PULPROG              zg30
TD                  65536
SOLVENT             CDCl3
NS                     64
DS                      0
SWH             8223.685 Hz
FIDRES          0.125483 Hz
AQ             3.9846387 sec
RG                    144
DW                 60.800 usec
DE                   8.00 usec
TE                  298.6 K
D1             1.00000000 sec
TD0                     1

======== CHANNEL f1 ========
NUC1                   1H
P1                  11.15 usec
PL1                 -3.00 dB
SFO1          400.1524711 MHz

F2 - Processing parameters
SI                  32768
SF            400.1500000 MHz
WDW                    GM
SSB                     0
LB                  -0.20 Hz
GB                    0.2
PC                   1.00
```

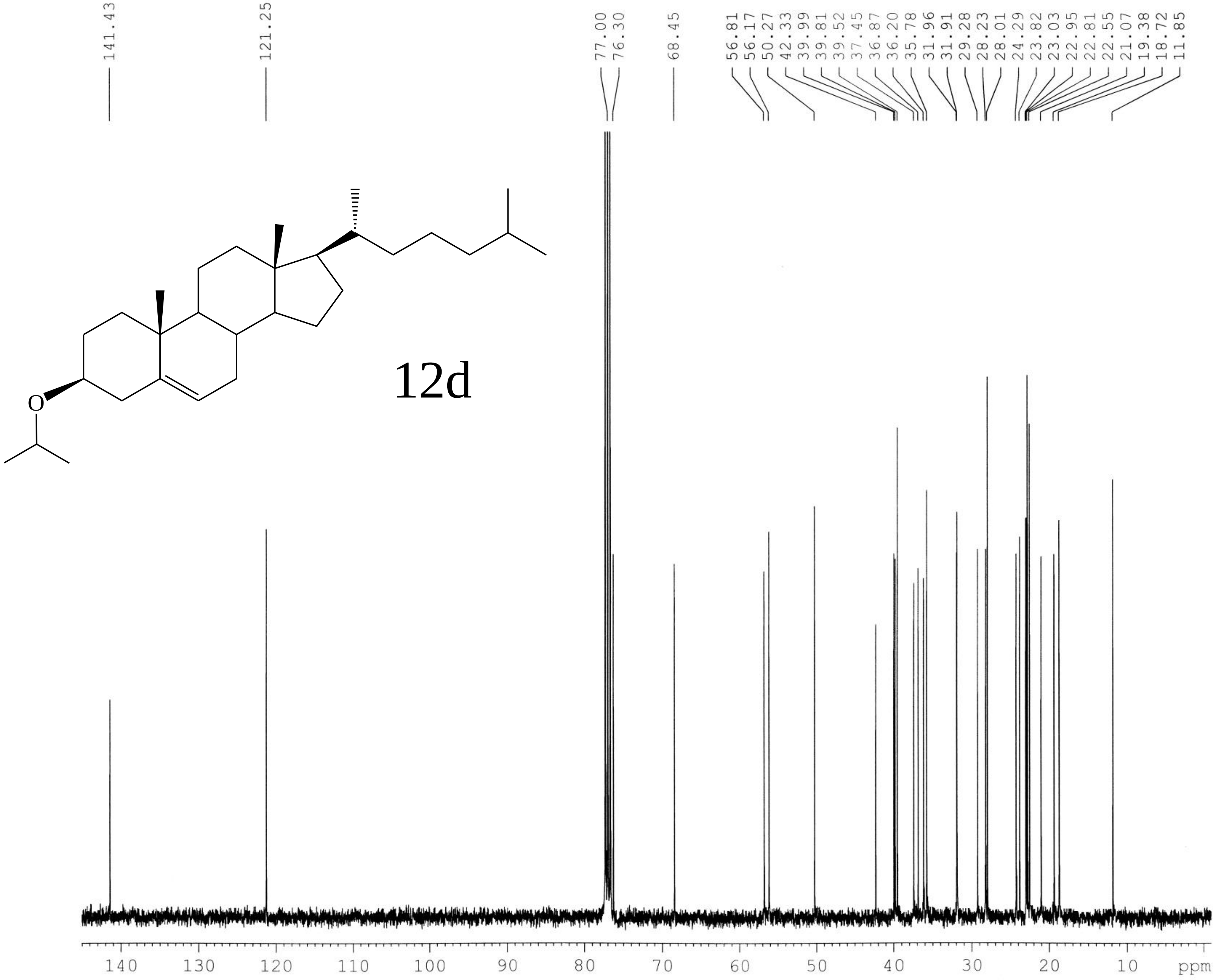

Current Data Parameters
NAME AT eterizopropylowyw3
EXPNO 2
PROCNO 1

F2 - Acquisition Parameters
Date_ 20140425
Time 16.16
INSTRUM spect
PROBHD 5 mm PABBO BB-
PULPROG zgpg30
TD 65536
SOLVENT CDCl3
NS 768
DS 4
SWH 27173.912 Hz
FIDRES 0.414641 Hz
AQ 1.2059124 sec
RG 2050
DW 18.400 usec
DE 6.00 usec
TE 299.1 K
D1 2.00000000 sec
d11 0.03000000 sec
DELTA 1.89999998 sec
TD0 1

======== CHANNEL f1 ========
NUC1 13C
P1 12.25 usec
PL1 -1.00 dB
SFO1 100.6288660 MHz

======== CHANNEL f2 ========
CPDPRG2 waltz16
NUC2 1H
PCPD2 100.00 usec
PL2 -3.00 dB
PL12 13.65 dB
PL13 18.00 dB
SFO2 400.1516006 MHz

F2 - Processing parameters
SI 32768
SF 100.6177982 MHz
WDW EM
SSB 0
LB 1.00 Hz
GB 0
PC 1.40

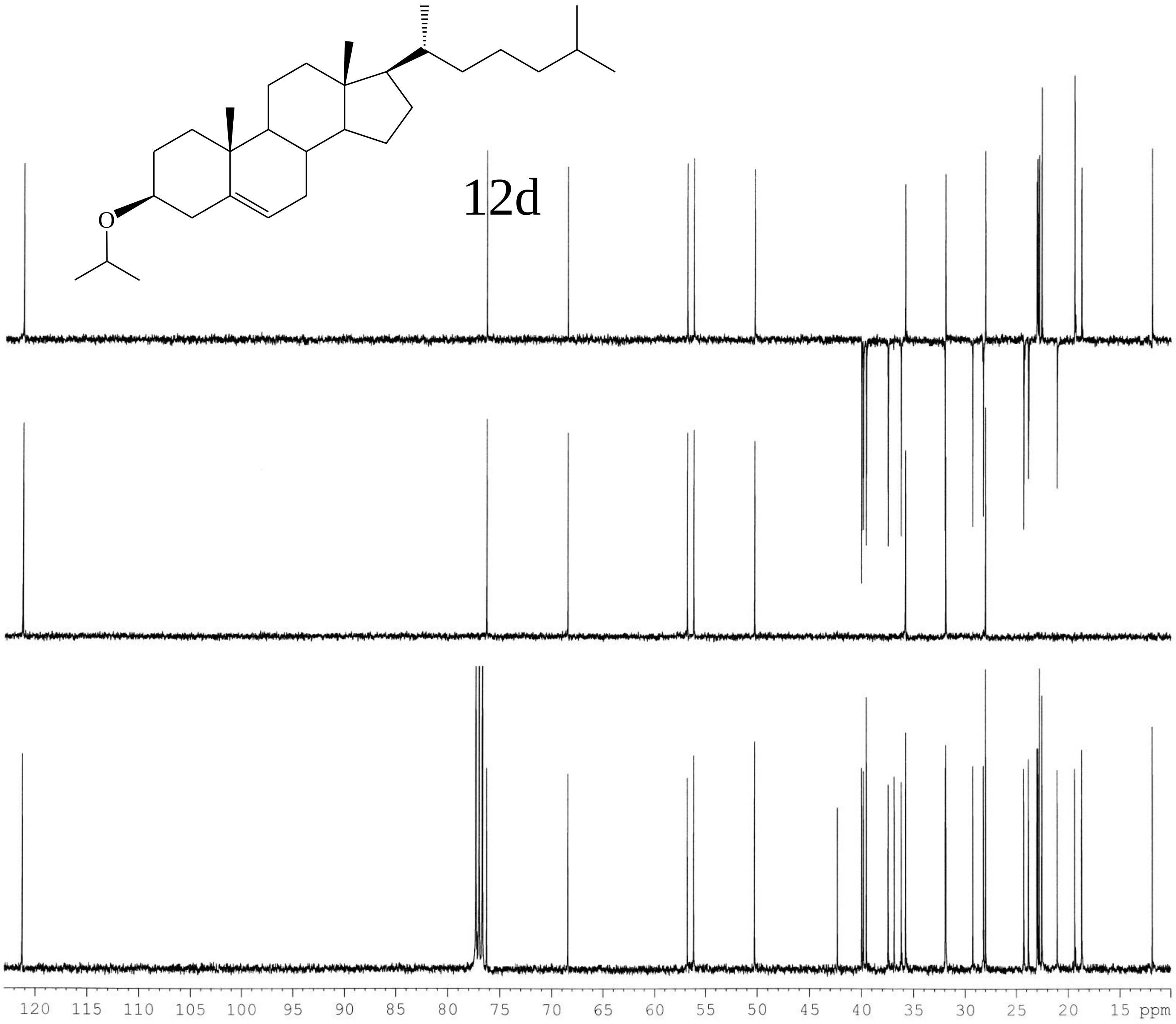

Current Data Parameters
NAME AT eterizopropylowyw3
EXPNO 4
PROCNO 1

F2 - Acquisition Parameters
Date_ 20140425
Time 17.28
INSTRUM spect
PROBHD 5 mm PABBO BB-
PULPROG dept135
TD 65536
SOLVENT CDCl3
NS 320
DS 4
SWH 24038.461 Hz
FIDRES 0.366798 Hz
AQ 1.3631988 sec
RG 2050
DW 20.800 usec
DE 6.00 usec
TE 299.4 K
CNST2 145.0000000
D1 2.00000000 sec
d2 0.00344828 sec
d12 0.00002000 sec
DELTA 0.00001560 sec
TD0 1

======== CHANNEL f1 ========
NUC1 13C
P1 12.25 usec
p2 24.50 usec
PL1 -1.00 dB
SFO1 100.6278593 MHz

======== CHANNEL f2 ========
CPDPRG2 waltz16
NUC2 1H
P3 14.70 usec
p4 29.40 usec
PCPD2 100.00 usec
PL2 -3.00 dB
PL12 13.65 dB
SFO2 400.1516006 MHz

F2 - Processing parameters
SI 32768
SF 100.6177983 MHz
WDW EM
SSB 0
LB 1.00 Hz
GB 0
PC 1.40

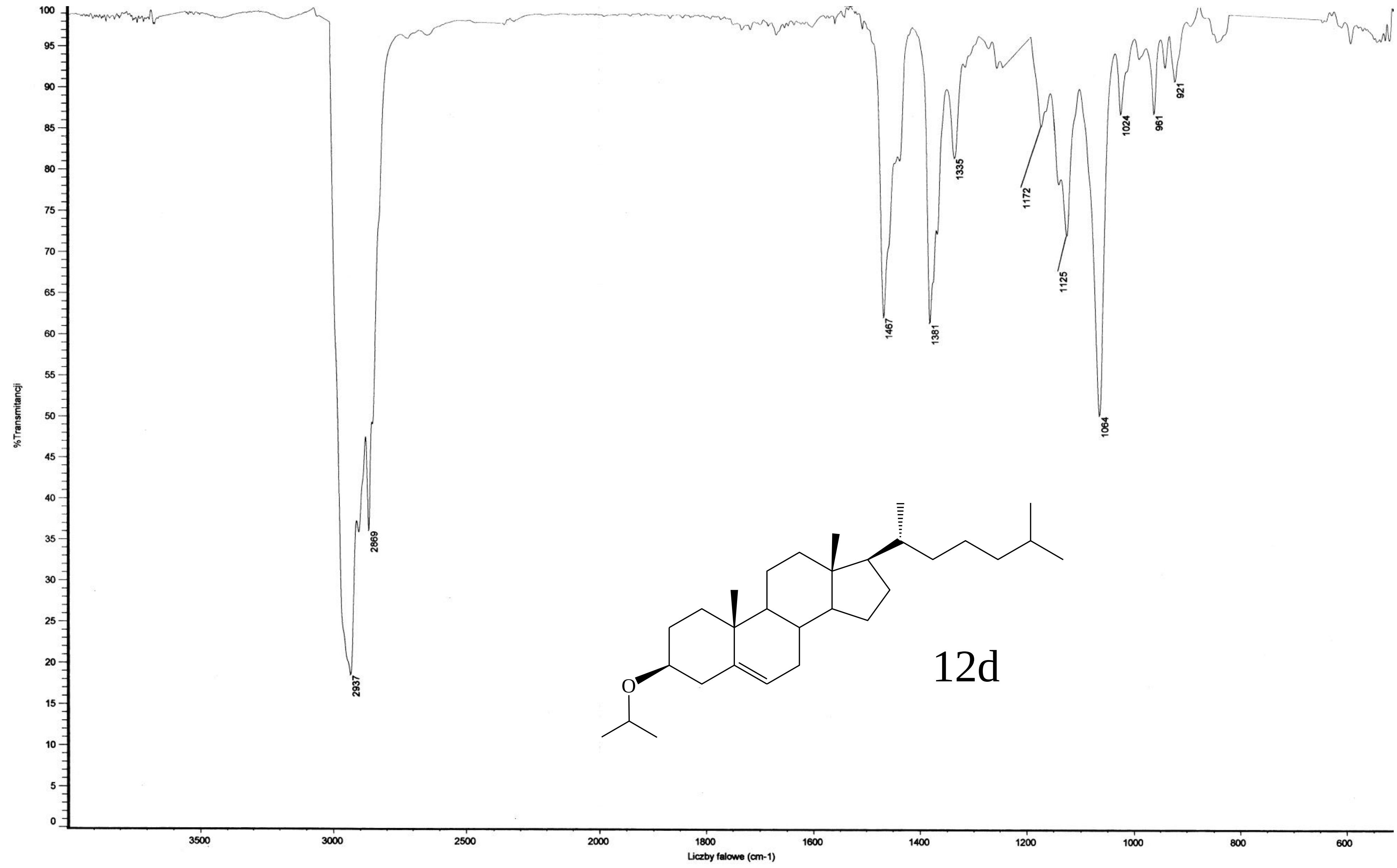

%Transmitancji
Liczby falowe (cm-1)
100
95
90
85
80
75
70
65
60
55
50
45
40
35
30
25
20
15
10
5
0
3500
3000
2500
2000
1800
1600
1400
1200
1000
800
600
2937
2869
1467
1381
1335
1172
1125
1064
1024
961
921
O
12d

J. Morzycki
eter izopropylowy w3
ub_jm1100 54 (4.396) Cm (54:72-5:8)

AUTOSPEC

14-Apr-2014 10:30:58
operator: Malgorzata Grela
Magnet EI+
5.01e3

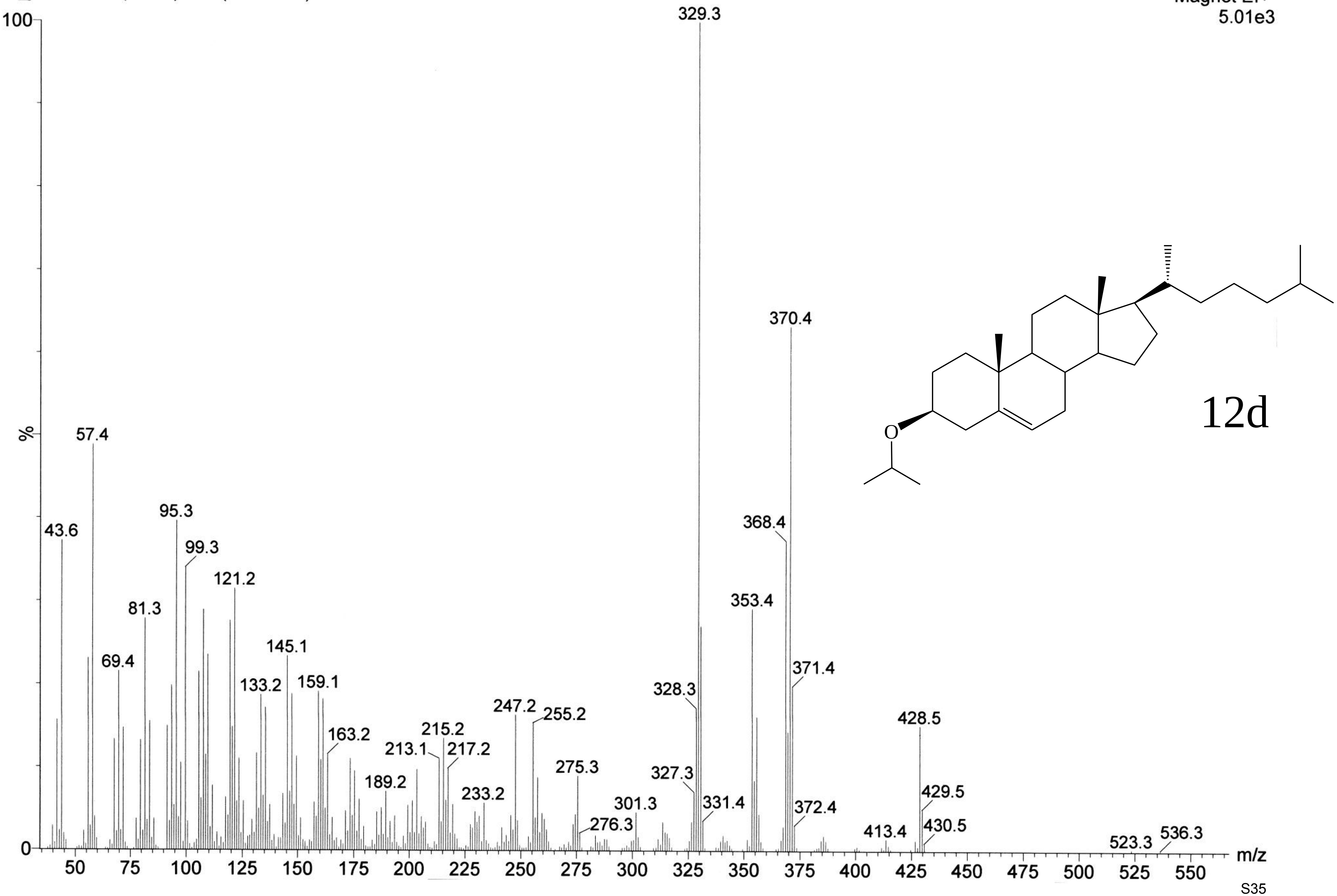

12d
